# Real-world costs of obesity-related complications over eight years: a US retrospective cohort study in 28,500 individuals

**DOI:** 10.1038/s41366-023-01376-4

**Published:** 2023-09-18

**Authors:** Jonathan Pearson-Stuttard, Tania Banerji, Silvia Capucci, Elisabeth de Laguiche, Mads D. Faurby, Christiane Lundegaard Haase, Kasper Sommer Matthiessen, Aimee M. Near, Jenny Tse, Xiaohui Zhao, Marc Evans

**Affiliations:** 1 Lane Clark & Peacock LLP, London, UK; 2 Department of Epidemiology and Biostatistics, School of Public Health, Imperial College London, London, UK; 3 IQVIA, Durham, NC USA; 4 Novo Nordisk A/S, Søborg, Denmark; 5 University Hospital, Llandough, Penarth, Cardiff UK

**Keywords:** Epidemiology, Disease prevention, Health policy, Risk factors

## Abstract

**Background:**

Obesity-related complications (ORCs) are associated with high costs for healthcare systems. We assessed the relationship between comorbidity burden, represented by both number and type of 14 specific ORCs, and total healthcare costs over time in people with obesity in the USA.

**Methods:**

Adults (≥ 18 years old) identified from linked electronic medical records and administrative claims databases, with a body mass index measurement of 30–< 70 kg/m^2^ between 1 January 2007 and 31 March 2012 (earliest measurement: index date), and with continuous enrolment for ≥ 1 year pre index (baseline year) and ≥ 8 years post index, were included. Individuals were grouped by type and number of ORCs during the pre-index baseline year. The primary outcome was annual total adjusted direct per-person healthcare costs.

**Results:**

Of 28,583 included individuals, 12,686 had no ORCs, 7242 had one ORC, 4180 had two ORCs and 4475 had three or more ORCs in the baseline year. Annual adjusted direct healthcare costs increased with the number of ORCs and over the 8-year follow-up. Outpatient costs were the greatest contributor to baseline annual direct costs, irrespective of the number of ORCs. For specific ORCs, costs generally increased gradually over the follow-up; the largest percentage increases from year 1 to year 8 were observed for chronic kidney disease (+ 78.8%) and type 2 diabetes (+ 47.8%).

**Conclusions:**

In a US real-world setting, the number of ORCs appears to be a cost driver in people with obesity, from the time of initial obesity classification and for at least the following 8 years.

## Introduction

Over the past four decades, the worldwide prevalence of obesity has nearly tripled, making obesity a major global public health issue [1]. In the USA, more than 40% of adults were estimated to have obesity in 2017–2018 [2]. The prevalence of obesity is increasing [2] and is expected to continue to rise, and the COVID-19 pandemic is likely to have further increased obesity rates in children and adults [3, 4]. It is estimated that by 2030 approximately 50% of adults in the USA will have obesity, and nearly 25% of adults will have class II or class III obesity (body mass index [BMI] > 35 kg/m^2^) [5, 6]. The prevalence of obesity in Europe is also increasing [7]; however, it is generally lower than in the USA, with an estimated average obesity prevalence of 24% across nine EU countries in 2018 [8].

Obesity is associated with a high economic burden, with an estimated 7.9% of US national medical expenditure devoted to treating obesity and related comorbidities in adults in 2015 [9]. According to a meta-analysis of 12 US studies published between 2008 and 2012, annual medical care costs attributable to obesity during this time were $1901 per person (in 2014 $), which corresponded to an annual total of $149.4 billion nationwide [10]. Compared with people without obesity, people living with obesity have an increased risk of cardiovascular (CV), metabolic and other complications, such as hypertension, dyslipidaemia, type 2 diabetes (T2D) and osteoarthritis (OA), as well as an increased risk for CV mortality [11]. These obesity-related complications (ORCs) contribute substantially to the economic burden of obesity [12, 13].

Previous studies have estimated costs associated with ORCs in the USA. A 2015 study by Li et al., assessing the economic burden of 21 ORCs using a database of electronic health records in people with and without obesity, identified hypertensive diseases, dyslipidaemia and OA as the costliest ORCs in people with obesity, each accounting for annual incremental costs of more than $23 million per year per 100,000 individuals versus people without any comorbidity [14]. However, this study used a regional database with a primarily White population (97%), and results may not be generalisable to the US population. Because the US population is highly heterogenous, it is important to gain evidence from large national databases to better understand the external validity of the results. In a study from 2021 by Divino et al., which quantified direct medical costs by BMI class of 13 ORCs over 1 year using linked electronic health records and claims databases, OA of the knee and heart failure (HF) with preserved ejection fraction were identified as the ORCs with the highest costs overall and with the biggest difference between healthy weight (BMI 18.5–24.9 kg/m^2^) and class III obesity (BMI ≥ 40 kg/m^2^) [15]. For these ORCs, mean total 1-year complication-specific costs per person with obesity ranged between $3719 and $4518 for OA of the knee and between $2855 and $4658 for HF with preserved ejection fraction. However, costs were primarily assessed after a relatively short follow-up of 1 year, with limited data on cost progression (maximum follow-up of 3 years).

Due to the wide range of ORCs, there is heterogeneity in the unmet needs and disease burden of people living with obesity. Identifying people with obesity who have the greatest burden of disease will be important to target preventative services and weight management approaches in a proportionate and effective manner. Analysis of nationwide longitudinal data on healthcare costs for people with obesity and ORCs is important to understand future implications for healthcare spending. In this retrospective cohort study of individuals with obesity in the USA, we aimed to examine the association between comorbidity burden, represented by both the number and the type of ORCs, and total healthcare costs over time.

## Materials and methods

### Data source

This study used linked data from the IQVIA Ambulatory Electronic Medical Records (AEMR) database [16] and the IQVIA PharMetrics® Plus database [17]. The AEMR database comprises over 80 million patient records starting from 2006, collected from 100,000 physicians. Key demographic and clinical variables can be linked to therapeutic outcomes and to prescriptions, diagnoses or hospital metrics. PharMetrics Plus is an aggregated database that includes adjudicated claims for more than 210 million unique patients across the USA. It provides a longitudinal overview of inpatient and outpatient services, prescription and office/outpatient-administered drugs, costs and detailed enrolment information. Data from both databases are compliant with the Health Insurance Portability and Accountability Act to protect patient privacy.

The Office for Human Research Protection under the US Department of Health and Human Services does not consider research of fully de-identified information to involve human subjects. This study was a retrospective analysis of secondary de-identified data and was not considered to be human subject research; therefore, ethical approval was not required.

### Study design and population

This was a retrospective cohort study. The index date was the date of first weight record for which the BMI was ≥ 30.0 kg/m^2^ and < 70.0 kg/m^2^ during the index period (1 January 2007 to 31 March 2012). BMI values at index were determined from available BMI records or calculated from available height and weight data in the AEMR during the index period. For inclusion, individuals were required to be ≥ 18 years old at the index date, to have continuous enrolment for ≥ 1 year pre index (baseline year) and ≥ 8 years post index (follow-up), and to have linkable data in the PharMetrics Plus database. Owing to the enrolment requirements, the full study period was 1 January 2006 to 31 March 2020.

Individuals were excluded from the study if, in the baseline year, they had one or more BMI value in AEMR or one or more obesity diagnosis code in PharMetrics Plus corresponding to their obesity class at index or a higher class. Individuals with invalid/missing age, sex, region or health plan enrolment dates were excluded, as were those who were pregnant in the baseline year. Those with a diagnosis of cancer (except non-melanoma skin cancer) at any point during all the available time of continuous enrolment in the pre-index period were also excluded. Finally, individuals with enrolment in a Medicare cost or State Children’s Health Insurance Program plan, and those with implausible changes in BMI (i.e., > 20% change in BMI within a 30-day period or > 20% average monthly change in BMI for measurements > 30 days apart) were also excluded.

### Obesity-related complications (ORCs) and comorbidities of interest

ORCs and comorbidities were identified via International Classification of Diseases Version 9 and 10 clinical modification diagnosis codes (ICD-9-CM and ICD-10-CM) during the baseline period using data from PharMetrics Plus. A full list of ICD codes is available in Table S1. ORCs were obstructive sleep apnoea, HF, urinary incontinence, OA of the knee, T2D, prediabetes, asthma, psoriasis, gastro-oesophageal reflux disease, hypertension, dyslipidaemia, musculoskeletal pain, chronic kidney disease (CKD; including kidney failure) and atherosclerotic cardiovascular disease (ASCVD). ASCVD included cerebrovascular disease, ischaemic heart disease and peripheral artery disease. These ORCs were previously identified as conditions with an established association with obesity and were the basis of the 2021 publication by Divino et al. determining 1-year complication-specific costs [15]. The following ORCs were of particular interest and costs were investigated in detail: T2D, HF, CKD and OA of the knee. In addition, the following conditions or group of conditions were of interest: established CVD, ≥ 2 ORCs, ≥ 3 ORCs and high CV risk. Established cardiovascular disease (CVD) included ASCVD, HF, cardiomyopathies, deep vein thrombosis and pulmonary embolism, cardiac arrest, atrial fibrillation and flutter, and atherosclerosis. Individuals at high risk for CV complications were defined as those with at least two CV risk factors out of hypertension, dyslipidaemia and T2D or prediabetes.

### Outcomes

The primary outcome was total direct per-person healthcare costs at baseline, in each year of follow-up and averaged over the 8 years of follow-up, derived from PharMetrics Plus. Direct healthcare costs included the reimbursed amount paid by payers combined with the patient out-of-pocket cost (e.g., co-pay, co-insurance). All costs were converted to 2019 US dollars using the medical component of the Consumer Price Index. Costs were calculated on a per-person basis and were averaged across each cohort; cohorts were defined by the number of ORCs (0, 1, 2 or ≥ 3) or the presence/absence of specific ORCs or comorbidities. In addition, the following costs were evaluated in the baseline year and annually during follow-up: inpatient (hospitalisation), outpatient medical care (emergency department [ED] and other outpatient medical care reported separately) and outpatient drug costs (pharmacy costs). Data are presented for the full population and by obesity class at index (class I: BMI ≥ 30.0–< 35.0 kg/m^2^; class II: BMI ≥ 35.0–< 40.0 kg/m^2^; class III: BMI ≥ 40.0–< 70.0 kg/m^2^). ORC cohorts are not mutually exclusive and therefore per-patient costs for specific ORCs are not additive.

### Statistical analyses

Both adjusted and observed costs are presented. Adjusted costs were estimated from generalised estimating equation models with a gamma distribution and log link to predict total annual costs for each cohort or subgroup at each time point from years 1 to 8. Each model included specific ORC(s) (yes vs. no) or number of ORCs (≥ 3, 2, 1 vs. 0), in addition to obesity class (III, II vs. I), age group, sex, region, year of cost; and interactions: age group*sex, age group*region, and ORC(s)*obesity class*year of cost. Baseline Charlson Comorbidity Index category, pre-index cost and ORCs other than the one being assessed were not included in the model due to high collinearity across different comorbidities and collinearity with age group. Analyses were conducted using SAS® Release 9.4 (SAS Institute Inc., Cary, NC, USA).

## Results

### Study population and baseline characteristics

A total of 28,583 individuals met all inclusion criteria (Table S2). At index, 17,892 (62.6%) individuals had class I obesity, 6550 (22.9%) had class II obesity and 4141 (14.5%) had class III obesity. More than half of the study population had ≥ 1 ORCs, and approximately one-third (30.3%) had ≥2 ORCs. Overall, 12,686 (44.4%) had no evidence of ORCs, 7242 (25.3%) had one ORC, 4180 (14.6%) had two ORCs and 4475 (15.7%) had ≥3 ORCs. In the groups with ≥2 or ≥3 ORCs, the most prevalent ORCs were hypertension (67.5% and 81.7%, respectively), musculoskeletal pain (53.1% and 59.8%) and dyslipidaemia (51.7% and 65.9%).

Baseline characteristics by number of ORCs are shown in Table 1; baseline characteristics for individuals with specific ORCs are shown in Table S3. The mean age of the full population was 45.6 years (standard deviation [SD] 10.4). The mean age was higher for those with more ORCs, ranging from 43.1 (SD 10.6) years in the group without ORCs to 51.7 (SD 8.9) years in the group with ≥ 3 ORCs. Less than half of included individuals were women across all groups. In all ORC groups, about two-thirds of the study population were White and only a small percentage (< 5%) were African American. The proportion of individuals from the Midwestern USA decreased with the number of ORCs, whereas the proportion of individuals from the Southern USA increased with the number of ORCs. A small proportion of individuals was from the Western USA (5.0%–6.2%). Most individuals were insured commercially or self-insured.

**Table 1:** Baseline characteristics of individuals with obesity, by number of ORCs.

Baseline characteristics	No ORCs *n* = 12,686	1 ORC *n* = 7242	2 ORCs *n* = 4180	≥ 3 ORCs *n* = 4475
**Age, years, mean (SD)**	43.1 (10.6)	44.8 (9.8)	48.3 (9.4)	51.7 (8.9)
**Women,** ***n*** **(%)**	5737 (45.2)	3469 (47.9)	1945 (46.5)	2004 (44.8)
**Region,** ***n*** **(%)**				
Northeast	4426 (34.9)	2579 (35.6)	1475 (35.3)	1451 (32.4)
Midwest	4157 (32.8)	2169 (30.0)	1098 (26.3)	1053 (23.5)
South	3319 (26.2)	2074 (28.6)	1378 (33.0)	1746 (39.0)
West	784 (6.2)	420 (5.8)	229 (5.5)	225 (5.0)
**Race/ethnicity,** ***n*** **(%)**				
White	8393 (66.2)	4888 (67.5)	2855 (68.3)	3109 (69.5)
African American	462 (3.6)	289 (4.0)	194 (4.6)	196 (4.4)
Asian	84 (0.7)	32 (0.4)	27 (0.6)	19 (0.4)
Hispanic	88 (0.7)	39 (0.5)	29 (0.7)	22 (0.5)
Unknown	3659 (28.8)	1994 (27.5)	1075 (25.7)	1129 (25.2)
**Payer type,** ***n*** **(%)**				
Commercial	6979 (55.0)	3866 (53.4)	2204 (52.7)	2288 (51.1)
Medicaid	43 (0.3)	23 (0.3)	17 (0.4)	3 (0.1)
Medicare Risk	39 (0.3)	41 (0.6)	52 (1.2)	134 (3.0)
Self-insured	5619 (44.3)	3311 (45.7)	1907 (45.6)	2050 (45.8)
Unknown	6 (< 0.1)	1 (< 0.1)	0 (0)	0 (0)
**CCI score, mean (SD)**	0.0 (0.2)	0.2 (0.5)	0.4 (0.6)	1.0 (1.1)

*CCI* Charlson Comorbidity Index, *ORC* obesity-related complication, *SD* standard deviation.

### Annual costs and cost progression by number of ORCs

**Fig. 1:**
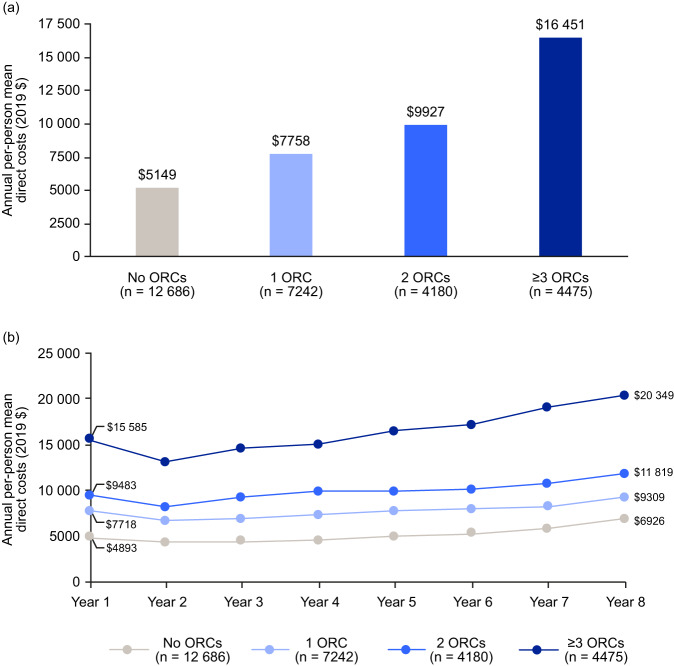
Adjusted mean annual total all-cause healthcare costs for individuals with obesity by number of ORCs. Costs are **a** averaged over 8 years of follow-up, and **b** shown per year of follow-up. *ORC* obesity-related complication.

Mean adjusted annual direct healthcare costs, averaged over 8 years of follow-up, were higher for individuals with ORCs than those without ORCs and increased with the number of ORCs (no ORCs: $5149; ≥ 3 ORCs: $16,451; Fig. 1A). Over time, costs increased in all groups (Fig. 1B). Annual costs per person increased from $4893 in year 1 to $6926 in year 8 in the group without ORCs (41.5% increase); from $7718 to $9309 in the group with one ORC (20.6%); and from $9483 to $11,819 in the group with two ORCs (24.6%). The ‘≥ 3 ORCs’ group had the highest costs in every year, and costs increased from $15,585 in year 1 to $20,349 in year 8 (30.6%). From year 2 to year 8, the most pronounced cost increase was in the group with ≥ 3 ORCs. Observed mean annual costs by number of ORCs were also recorded and support the trend seen with adjusted costs (Fig. S1).

### Annual costs for specific ORCs

**Fig. 2:**
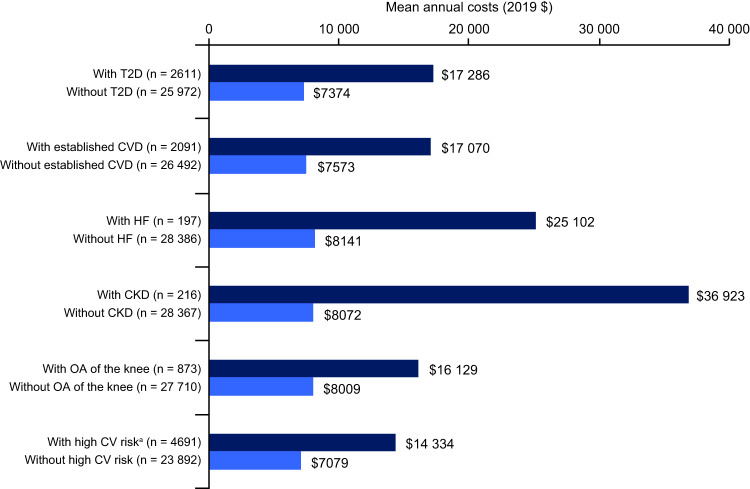
Adjusted mean annual total all-cause healthcare costs for individuals with obesity, averaged over 8 years follow-up and stratified by the presence (‘with’ group) or absence (‘without’ group) of specific ORCs or numbers of ORC. CKD chronic kidney disease, CV cardiovascular, CVD cardiovascular disease, HF heart failure, OA osteoarthritis, ORC obesity-related complication, T2D type 2 diabetes. ^a^High CV risk: ≥ 2 risk factors out of hypertension, dyslipidaemia and T2D/prediabetes. The ‘without’ group has < 2 CV risk factors.

For all groups, adjusted annual healthcare costs, averaged over 8 years of follow-up, were higher for individuals with any given ORC than for those without this ORC (Fig. 2). Costs approximately doubled for ‘with’ versus ‘without’ ORC groups for T2D, established CVD and OA of the knee, and for those at high CV risk. Costs for individuals with HF and CKD were substantially (3–4 times) higher than costs for those that did not have the respective ORC in the baseline year (HF, $25,102 vs. $8141; CKD, $36,923 vs. $8072). However, it is noteworthy that very few individuals had these ORCs (HF, *n* = 197; CKD, *n* = 216; each corresponding to < 1% of the total population [*n* = 28,583]). Similar trends were found for observed annual healthcare costs, averaged over 8 years of follow-up (Fig. S2).

### Cost progression for specific ORCs

**Fig. 3:**
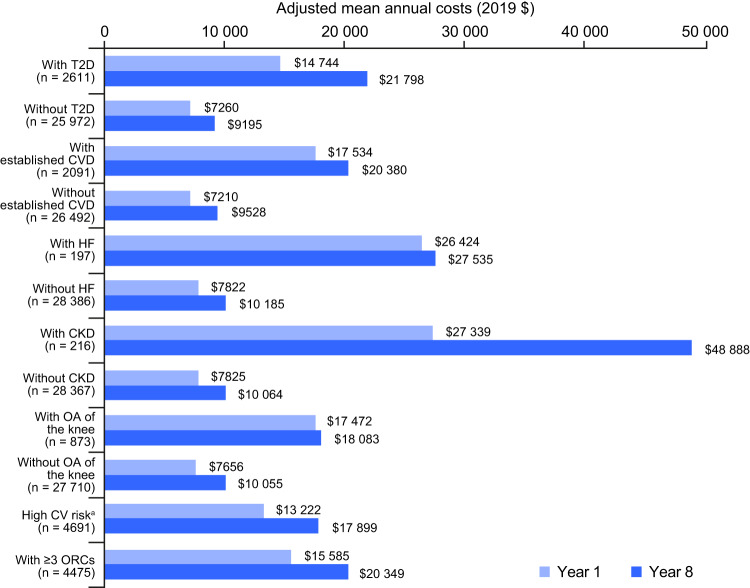
Adjusted mean total all-cause per-person healthcare costs at year 1 and at year 8 among individuals with obesity, stratified by specific ORCs. CKD chronic kidney disease, CVD cardiovascular disease, HF heart failure, OA osteoarthritis, ORC obesity-related complication, T2D type 2 diabetes. ^a^High CV risk: ≥ 2 risk factors out of hypertension, dyslipidaemia and T2D/prediabetes.

When cost progression was examined according to the presence or absence of a specific ORC, adjusted annual healthcare costs for specific ORCs generally increased from year 1 to year 8 for all groups (Fig. 3); data on observed costs support this pattern (Fig. S3). In line with the observed high averaged annual costs for CKD, costs associated with CKD were the highest of all ORCs of interest in year 1 ($27,339) and year 8 ($48,888), which corresponds to a 78.8% increase, the largest cost increase observed. Costs associated with T2D increased by 47.8%. Increases of approximately 30% were observed for individuals with ≥ 3 ORCs or for those at high CV risk. For individuals with established CVD, costs increased by 16.2%. Notable exceptions to increasing costs were HF and OA of the knee, for which costs were comparatively stable from year 1 to year 8 (4.2% and 3.5% increase, respectively); however, HF was associated with relatively high costs in year 1 and year 8 ($26,424 and $27,535, respectively).

Adjusted annual costs for each year of follow-up were also recorded (Table S4). For some ORC groups (e.g., T2D), costs increased steadily over time, whereas for other ORCs (e.g., OA of the knee), costs increased over time, but annual costs fluctuated. Observed costs (Table S5) showed the same pattern as adjusted costs.

Adjusted costs stratified by obesity class were calculated to assess the contribution of obesity severity to ORC-related costs (Table S6). For most ORCs, there were no notable differences in adjusted costs across obesity classes. The same was noted for observed costs by obesity class (Table S7).

### Cost drivers for specific ORCs

**Fig. 4:**
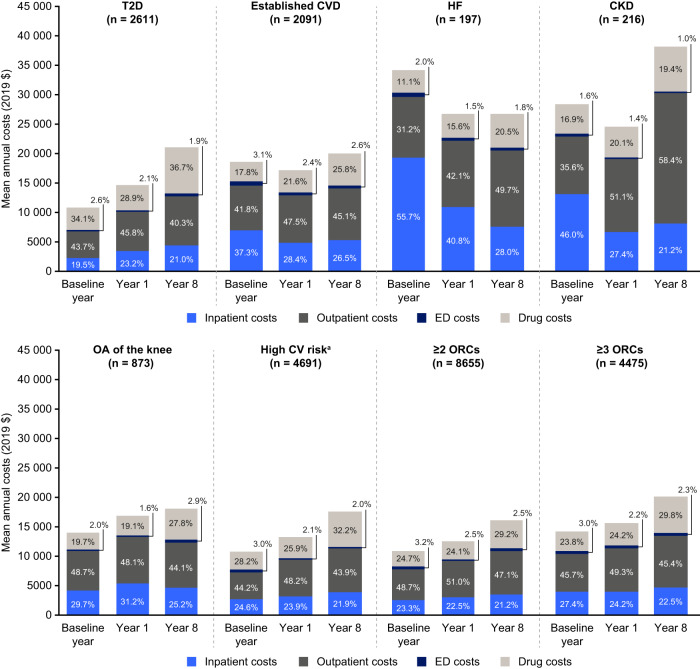
Observed mean inpatient, outpatient, ED and drug costs per person in the baseline year and in years 1 and 8. CKD chronic kidney disease, CVD cardiovascular disease, ED emergency department, HF heart failure, OA osteoarthritis, ORC obesity-related complication, T2D type 2 diabetes. ^a^High CV risk: at least two risk factors out of hypertension, dyslipidaemia and T2D/prediabetes.

To identify cost drivers, observed costs were used, because they provide a measure for the actual burden on patients and healthcare systems. Observed inpatient, outpatient and ED costs, as well as drug costs, contributing to total healthcare costs at baseline, in year 1 and year 8 are shown in Fig. 4, and costs for all years can be found in Table S8. For most ORCs, inpatient, outpatient and drug costs increased from baseline to year 8. Costs for visits to the ED were largely stable throughout the follow-up period and did not contribute markedly to total healthcare costs. Drug costs approximately doubled for the group with T2D (from $3832 at baseline to $7906 in year 8). A considerable increase in drug costs was also noted for individuals with established CVD (from $3434 to $5326), with OA of the knee (from $2848 to $5182), with high CV risk (from $3148 to $5819) and with ≥ 3 ORCs (from $3514 to $6124). Outpatient medical costs were generally the largest cost driver, constituting between 40.3% and 58.4% of total healthcare costs in year 8. At baseline, year 1 and year 8, outpatient costs were the largest cost contributor for all groups except those with HF and CKD at baseline, for whom inpatient costs constituted 46.0% or more of total healthcare costs at baseline. However, inpatient costs for these groups decreased and outpatient costs increased in subsequent years; outpatient costs for the group with CKD more than doubled over follow-up.

## Discussion

This study assessed cost progression over 8 years in a large US real-world cohort of individuals with obesity. Our study has three key findings. Firstly, the results suggest that the majority of people living with obesity have ORCs: more than 50% of individuals included in this study had been diagnosed with one or more ORCs at baseline. Secondly, costs associated with ORCs increase over time. Total direct healthcare costs increased over follow-up for most ORCs and were substantially higher for individuals with at least one ORC than for those without any ORCs. For each specific ORC, costs were higher for groups with the condition than without it. Costs also increased across obesity classes; however, for individuals with the same ORC, there were generally no notable differences in costs across obesity classes. It is noteworthy that across all obesity classes the bulk of cost incurred (> 60% of total costs) was healthcare system-based costs (i.e., outpatient, inpatient and ED costs). Although the excess cost burden of increasing BMI may not be apparent superficially, the additional burden is likely to exert significant capacity pressures on already under-resourced healthcare systems. Such considerations require further evaluation and represent an important value metric to be evaluated in relation to weight loss.

Thirdly, our results suggest that ORCs are driving the costs associated with obesity. Although cost drivers were not identified systematically, it appears that individuals with three or more ORCs incurred higher costs than those with fewer or no ORCs in every year throughout follow-up. The most prevalent comorbidities in the group of individuals with three or more ORCs were hypertension, dyslipidaemia and musculoskeletal pain; however, a previous retrospective analysis by Divino et al. (2021) quantifying complication-specific direct medical costs in the AEMR and PharMetrics Plus databases suggested that each of these conditions is not particularly costly in isolation [15]. Therefore, our data suggest that the presence of several ORCs results in an interaction effect, whereby the actual total costs are higher than the sum of the individual contributions. Importantly, no specific comorbidity emerged as a particular high-cost driver. Certain ORCs, in particular CKD and HF, were associated with high per-person costs, but very few individuals (< 1% for each) had been diagnosed with these ORCs in the baseline period. However, because the prevalence of HF and CKD is expected to increase over time in this patient population, preventative measures targeting CKD and HF are important to healthcare systems from a budget impact perspective. Previous US [15, 18] and global [19] studies have shown that costs increase with increasing BMI. In addition, a higher BMI is characterised by a higher comorbidity burden [20]. In our study, there were no striking differences in costs across obesity classes within specific ORC groups. It is therefore plausible that it is principally the number of ORCs, which is likely to be driven by having a high BMI, that contributes to the high overall costs associated with obesity.

Metabolic syndrome, a combination of several CV risk factors including obesity, dyslipidaemia, hyperglycaemia and hypertension, has been associated with an increased risk of developing diabetes and CVD, and of CVD mortality [21]. In this study, having two or more CV risk factors in addition to obesity was associated with increased total healthcare costs averaged over 8 years compared with having one risk factor or none. Costs for people at high CV risk were similar to those incurred by people with T2D. In line with our finding that more ORCs lead to higher costs, it is also apparent that the presence of multiple CV risk factors can be costly, even before another condition has developed.

Although this is an emerging scientific area, our findings are largely consistent with the published evidence to date. In a 2014 US study, there was a statistically significant increase in total costs per visit to an inpatient, outpatient or ED facility for people diagnosed with obesity and ORCs compared with people who had obesity only, with congestive HF showing the highest increase ($5275) among single comorbidities [12]. Similarly, a 2016 UK cohort study identified the presence of a comorbidity as the largest predictor of healthcare costs associated with obesity in primary care, causing a mean increase in annual patient costs of £1366 [13]. The 2021 study by Divino et al. identified OA of the knee and HF with preserved ejection fraction as the most costly of 13 ORCs examined, across all obesity classes [15]. An unexpected finding of our study was that outpatient costs were the main cost driver, contributing at least 40% of the total healthcare costs in years 1 to 8 across ORCs evaluated. This is in contrast to other studies from the USA [22] and the UK [23], in which inpatient costs accounted for the largest proportions of direct costs. However, in the study by Divino et al., outpatient care was also the main driver for overall costs [15]. It can be speculated that because people with obesity and ORCs in our study population have a large number of outpatient visits, they may be closely medically managed, which translates to fewer inpatient stays.

This was a large US-wide database study, including nearly 30,000 individuals with obesity and ORCs. The data have allowed a multi-faceted description of the burden that obesity imposes on health and healthcare systems, and the 8-year follow-up permitted a long-term analysis of ORC-related cost progression. However, the results are based on a US population of commercially insured individuals with a recorded BMI indicating obesity; as such, they may not be representative of uninsured or Medicare/Medicaid populations, and the results may not be fully generalisable to the entire US population or other countries. Furthermore, continuous database enrolment for the full study duration was an inclusion criterion, which may have led to cohort selection bias, whereby those included were healthy enough to be employed (and hence have insurance) and had not died within the study period. The study design required an interaction with the healthcare system in the index year, meaning that relatively higher costs in year 1 were observed, followed by a reduction in costs in the year after. A further limitation is that ORC severity, lifestyle factors, socioeconomic status/deprivation and level of education were not available in the databases and could not be adjusted for. Race and ethnicity were balanced across ORC cohorts, and therefore it is unlikely that the main conclusions are confounded by ethnic differences between cohorts. Further research is necessary to evaluate the impact of ORCs in populations not represented in this study, particularly elderly individuals without commercial insurance and those covered by fee-for-service Medicare and Medicaid. Another consideration is that a broader set of conditions, including many cancers [24], are associated with obesity, and future work could capture the total health need of people living with obesity and associated health system costs.

## Conclusion

In a US real-world setting of commercially insured people with obesity, there was a link between the number of ORCs and total healthcare costs, irrespective of obesity class, from the time of initial obesity classification and for at least the following 8 years. The main cost driver associated with obesity appears to be the number of ORCs, not the type. The substantial increase in costs over time in people with obesity and ORCs emphasises the need for early and effective weight management, including targeted interventions and care pathways, to prevent the onset of ORCs, irrespective of obesity severity. Focusing on managing and preventing ORCs might help to alleviate the burden of obesity for patients and healthcare systems alike.

### Supplementary information

R2R edits for supplementary materials

## Data Availability

This was a database analysis using IQVIA Ambulatory Electronic Medical Records (AEMR) database and IQVIA PharMetrics® Plus closed claims data obtained under license from IQVIA Inc. Individual patient data cannot be publicly shared. The authors confirm that the data supporting the findings of this study are available within the article and its supplementary materials. Interested researchers may reach out to IQVIA Inc. for discussing feasibility of accessing the study database.

## References

[1] World Health Organization. Obesity and overweight. 2021. https://www.who.int/news-room/fact-sheets/detail/obesity-and-overweight. Accessed 1 June 2022.

[2] Centers for Disease Control and Prevention. Prevalence of obesity and severe obesity among adults: United States, 2017–2018. 2020. https://www.cdc.gov/nchs/products/databriefs/db360.htm. Accessed 6 January 2022.

[3] Lin AL, Vittinghoff E, Olgin JE, Pletcher MJ, Marcus GM (2021). Body weight changes during pandemic-related shelter-in-place in a longitudinal cohort study. JAMA Netw Open.

[4] Lange SJ, Kompaniyets L, Freedman DS, Kraus EM, Porter R, Blanck HM (2021). Longitudinal trends in body mass index before and during the COVID-19 pandemic among persons aged 2–19 years — United States, 2018–2020. MMWR Morb Mortal Wkly Rep.

[5] Ward ZJ, Bleich SN, Cradock AL, Barrett JL, Giles CM, Flax C (2019). Projected U.S. state-level prevalence of adult obesity and severe obesity. N Engl J Med.

[6] Centers for Disease Control and Prevention. Defining adult overweight & obesity. 2021. https://www.cdc.gov/obesity/basics/adult-defining.html. Accessed 5 January 2022.

[7] Peralta M, Ramos M, Lipert A, Martins J, Marques A (2018). Prevalence and trends of overweight and obesity in older adults from 10 European countries from 2005 to 2013. Scand J Public Health.

[8] OECD/European Union. Obesity among adults. Health at a Glance: Europe 2020: State of Health in the EU Cycle: OECD Publishing, Paris; 2020.

[9] Biener A, Cawley J, Meyerhoefer C (2018). The impact of obesity on medical care costs and labor market outcomes in the US. Clin Chem.

[10] Kim DD, Basu A (2016). Estimating the medical care costs of obesity in the United States: systematic review, meta-analysis, and empirical analysis. Value Health.

[11] National Institute of Health. Managing overweight and obesity in adults: systematic evidence review from the obesity expert panel. 2013. https://www.nhlbi.nih.gov/sites/default/files/media/docs/obesity-evidence-review.pdf. Accessed 4 January 2022.

[12] Padula WV, Allen RR, Nair KV (2014). Determining the cost of obesity and its common comorbidities from a commercial claims database. Clin Obes.

[13] Gulliford MC, Charlton J, Booth HP, Fildes A, Khan O, Reddy M, et al. Chapter 5. Costs associated with obesity in primary care. Costs and outcomes of increasing access to bariatric surgery for obesity: cohort study and cost-effectiveness analysis using electronic health records. Southampton (UK): NIHR Journals Library (Health Services and Delivery Research, No. 4.17); 2016. 27253004

[14] Li Q, Blume SW, Huang JC, Hammer M, Ganz ML (2015). Prevalence and healthcare costs of obesity-related comorbidities: evidence from an electronic medical records system in the United States. J Med Econ.

[15] Divino V, Ramasamy A, Anupindi VR, Eriksen KT, Olsen AH, DeKoven M (2021). Complication-specific direct medical costs by body mass index for 13 obesity-related complications: a retrospective database study. J Manag Care Spec Pharm.

[16] IQVIA. US EMR – IQVIA Ambulatory EMR. 2020. https://www.iqvia.com/locations/united-states/library/fact-sheets/iqvia-ambulatory-emr-us. Accessed 30 March 2022.

[17] IQVIA. US claims – IQVIA PharMetrics Plus. 2022. https://www.iqvia.com/locations/united-states/library/fact-sheets/iqvia-pharmetrics-plus. Accessed 19 May 2022.

[18] Ward ZJ, Bleich SN, Long MW, Gortmaker SL (2021). Association of body mass index with health care expenditures in the United States by age and sex. PLoS One.

[19] Kent S, Fusco F, Gray A, Jebb SA, Cairns BJ, Mihaylova B (2017). Body mass index and healthcare costs: a systematic literature review of individual participant data studies. Obes Rev.

[20] Guh DP, Zhang W, Bansback N, Amarsi Z, Birmingham CL, Anis AH (2009). The incidence of co-morbidities related to obesity and overweight: A systematic review and meta-analysis. BMC Public Health.

[21] Dekker JM, Girman C, Rhodes T, Nijpels G, Stehouwer CD, Bouter LM (2005). Metabolic syndrome and 10-year cardiovascular disease risk in the Hoorn Study. Circulation..

[22] Cawley J, Biener A, Meyerhoefer C, Ding Y, Zvenyach T, Smolarz BG (2021). Direct medical costs of obesity in the United States and the most populous states. J Manag Care Spec Pharm.

[23] Tigbe WW, Briggs AH, Lean MEJ (2013). A patient-centred approach to estimate total annual healthcare cost by body mass index in the UK Counterweight programme. Int J Obes.

[24] Colditz GA, Peterson LL (2018). Obesity and cancer: evidence, impact, and future directions. Clin Chem.

