# Supplementary material for: Real-world costs of obesity-related complications over eight years: a US retrospective cohort study in 28,500 individuals
Source: Int J Obes (Lond). 2023 Sep 18;47(12):1239–46. doi: 10.1038/s41366-023-01376-4 (PMC10663144; doi:10.1038/s41366-023-01376-4)
Supplement: Supplementary file 1 — R2R edits for supplementary materials [file 41366_2023_1376_MOESM1_ESM.pdf]

Supplementary information

Real-world costs of obesity-related complications over eight years: a US retrospective cohort study in 28 500 individuals

Jonathan Pearson-Stuttard[1,2], Tania Banerji[3], Silvia Capucci[4], Elisabeth de Laguiche[4], Mads D. Faurby[4], Christiane Lundegaard Haase[4], Kasper Sommer Matthiessen[4], Aimee Near[3], Jenny Tse[3], Xiaohui Zhao[3], Marc Evans[5]

Affiliations:

[1]Lane Clark & Peacock LLP, London, UK

[2]Department of Epidemiology and Biostatistics, School of Public Health, Imperial College London, London, UK

[3]IQVIA, Durham, North Carolina, USA

[4]Novo Nordisk A/S, Søborg, Denmark

[5]University Hospital, Llandough, Penarth, Cardiff, UK

Table S1 List of ICD-9-CM and ICD-10-CM diagnosis codes for ORCs of interest.

Condition	Diagnosis code	Diagnosis version	Diagnosis description
Asthma	493	ICD-9-CM	Asthma
	493.0	ICD-9-CM	Extrinsic asthma
	493.00	ICD-9-CM	Extrinsic asthma, unspecified
	493.01	ICD-9-CM	Extrinsic asthma with status asthmaticus
	493.02	ICD-9-CM	Extrinsic asthma, with (acute) exacerbation
	493.1	ICD-9-CM	Intrinsic asthma
	493.10	ICD-9-CM	Intrinsic asthma, unspecified
	493.11	ICD-9-CM	Intrinsic asthma with status asthmaticus
	493.12	ICD-9-CM	Intrinsic asthma, with (acute) exacerbation
	493.2	ICD-9-CM	Chronic obstructive asthma
	493.20	ICD-9-CM	Chronic obstructive asthma, unspecified
	493.21	ICD-9-CM	Chronic obstructive asthma with status asthmaticus
	493.22	ICD-9-CM	Chronic obstructive asthma, with (acute) exacerbation
	493.8	ICD-9-CM	Other forms of asthma
	493.81	ICD-9-CM	Exercise induced bronchospasm
	493.82	ICD-9-CM	Cough variant asthma
	493.9	ICD-9-CM	Asthma, unspecified
	493.90	ICD-9-CM	Asthma, unspecified type, unspecified
	493.91	ICD-9-CM	Asthma, unspecified type, with status asthmaticus
	493.92	ICD-9-CM	Asthma, unspecified type, with (acute) exacerbation
	J45	ICD-10-CM	Asthma
	J45.2	ICD-10-CM	Mild intermittent asthma
	J45.20	ICD-10-CM	Mild intermittent asthma, uncomplicated
	J45.21	ICD-10-CM	Mild intermittent asthma with (acute) exacerbation
	J45.22	ICD-10-CM	Mild intermittent asthma with status asthmaticus
	J45.3	ICD-10-CM	Mild persistent asthma
	J45.30	ICD-10-CM	Mild persistent asthma, uncomplicated

	J45.31	ICD-10-CM	Mild persistent asthma with (acute) exacerbation
	J45.32	ICD-10-CM	Mild persistent asthma with status asthmaticus
	J45.4	ICD-10-CM	Moderate persistent asthma
	J45.40	ICD-10-CM	Moderate persistent asthma, uncomplicated
	J45.41	ICD-10-CM	Moderate persistent asthma with (acute) exacerbation
	J45.42	ICD-10-CM	Moderate persistent asthma with status asthmaticus
	J45.5	ICD-10-CM	Severe persistent asthma
	J45.50	ICD-10-CM	Severe persistent asthma, uncomplicated
	J45.51	ICD-10-CM	Severe persistent asthma with (acute) exacerbation
	J45.52	ICD-10-CM	Severe persistent asthma with status asthmaticus
	J45.9	ICD-10-CM	Other and unspecified asthma
	J45.90	ICD-10-CM	Unspecified asthma
	J45.901	ICD-10-CM	Unspecified asthma with (acute) exacerbation
	J45.902	ICD-10-CM	Unspecified asthma with status asthmaticus
	J45.909	ICD-10-CM	Unspecified asthma, uncomplicated
	J45.99	ICD-10-CM	Other asthma
	J45.990	ICD-10-CM	Exercise induced bronchospasm
	J45.991	ICD-10-CM	Cough variant asthma
	J45.998	ICD-10-CM	Other asthma
ASCVD	A total of 867 codes were used to identify ASCVD. A full list can be requested from the authors.		
CKD	403	ICD-9-CM	Hypertensive chronic kidney disease
	403.0	ICD-9-CM	Hypertensive chronic kidney disease, malignant
	403.00	ICD-9-CM	Hypertensive chronic kidney disease, malignant, with chronic kidney disease stage I through stage IV, or unspecified
	403.01	ICD-9-CM	Hypertensive chronic kidney disease, malignant, with chronic kidney disease stage V or end stage renal disease
	403.1	ICD-9-CM	Hypertensive chronic kidney disease, benign
	403.10	ICD-9-CM	Hypertensive chronic kidney disease, benign, with chronic kidney disease stage I through stage IV, or unspecified

403.11	ICD-9-CM	Hypertensive chronic kidney disease, benign, with chronic kidney disease stage v or end stage renal disease
403.9	ICD-9-CM	Hypertensive chronic kidney disease, unspecified
403.90	ICD-9-CM	Hypertensive chronic kidney disease, unspecified, with chronic kidney disease stage I through stage IV, or unspecified
403.91	ICD-9-CM	Hypertensive chronic kidney disease, unspecified, with chronic kidney disease stage V or end stage renal disease
404	ICD-9-CM	Hypertensive heart and chronic kidney disease
404.0	ICD-9-CM	Hypertensive heart and chronic kidney disease, malignant
404.00	ICD-9-CM	Hypertensive heart and chronic kidney disease, malignant, without heart failure and with chronic kidney disease stage I through stage IV, or unspecified
404.01	ICD-9-CM	Hypertensive heart and chronic kidney disease, malignant, with heart failure and with chronic kidney disease stage I through stage IV, or unspecified
404.02	ICD-9-CM	Hypertensive heart and chronic kidney disease, malignant, without heart failure and with chronic kidney disease stage V or end stage renal disease
404.03	ICD-9-CM	Hypertensive heart and chronic kidney disease, malignant, with heart failure and with chronic kidney disease stage V or end stage renal disease
404.1	ICD-9-CM	Hypertensive heart and chronic kidney disease,benign
404.10	ICD-9-CM	Hypertensive heart and chronic kidney disease, benign, without heart failure and with chronic kidney disease stage I through stage IV, or unspecified
404.11	ICD-9-CM	Hypertensive heart and chronic kidney disease, benign, with heart failure and with chronic kidney disease stage I through stage IV, or unspecified
404.12	ICD-9-CM	Hypertensive heart and chronic kidney disease, benign, without heart failure and with chronic kidney disease stage V or end stage renal disease
404.13	ICD-9-CM	Hypertensive heart and chronic kidney disease, benign, with heart failure and chronic kidney disease stage V or end stage renal disease
404.9	ICD-9-CM	Hypertensive heart and chronic kidney disease, unspecified
404.90	ICD-9-CM	Hypertensive heart and chronic kidney disease, unspecified, without heart failure and with chronic kidney disease stage I through stage IV, or unspecified

404.91	ICD-9-CM	Hypertensive heart and chronic kidney disease, unspecified, with heart failure and with chronic kidney disease stage I through stage IV, or unspecified
404.92	ICD-9-CM	Hypertensive heart and chronic kidney disease, unspecified, without heart failure and with chronic kidney disease stage v or end stage renal disease
404.93	ICD-9-CM	Hypertensive heart and chronic kidney disease, unspecified, with heart failure and chronic kidney disease stage V or end stage renal disease
585	ICD-9-CM	Chronic kidney disease (CKD)
585.1	ICD-9-CM	Chronic kidney disease, stage I
585.2	ICD-9-CM	Chronic kidney disease, stage II (mild)
585.3	ICD-9-CM	Chronic kidney disease, stage III (moderate)
585.4	ICD-9-CM	Chronic kidney disease, stage IV (severe)
585.5	ICD-9-CM	Chronic kidney disease, stage V
585.6	ICD-9-CM	End stage renal disease
585.9	ICD-9-CM	Chronic kidney disease, unspecified
I12	ICD-10-CM	Hypertensive chronic kidney disease
I12.0	ICD-10-CM	Hypertensive chronic kidney disease with stage 5 chronic kidney disease or end stage renal disease
I12.9	ICD-10-CM	Hypertensive chronic kidney disease with stage 1 through stage 4 chronic kidney disease, or unspecified chronic kidney disease
I13	ICD-10-CM	Hypertensive heart and chronic kidney disease
I13.0	ICD-10-CM	Hypertensive heart and chronic kidney disease with heart failure and stage 1 through stage 4 chronic kidney disease, or unspecified chronic kidney disease
I13.1	ICD-10-CM	Hypertensive heart and chronic kidney disease without heart failure
I13.10	ICD-10-CM	Hypertensive heart and chronic kidney disease without heart failure, with stage 1 through stage 4 chronic kidney disease, or unspecified chronic kidney disease
I13.11	ICD-10-CM	Hypertensive heart and chronic kidney disease without heart failure, with stage 5 chronic kidney disease, or end stage renal disease
I13.2	ICD-10-CM	Hypertensive heart and chronic kidney disease with heart failure and with stage 5 chronic kidney disease, or end stage renal disease

	N18	ICD-10-CM	Chronic kidney disease (CKD)
	N18.1	ICD-10-CM	Chronic kidney disease, stage 1
	N18.2	ICD-10-CM	Chronic kidney disease, stage 2 (mild)
	N18.3	ICD-10-CM	Chronic kidney disease, stage 3 (moderate)
	N18.30	ICD-10-CM	Chronic kidney disease, stage 3 unspecified
	N18.31	ICD-10-CM	Chronic kidney disease, stage 3a
	N18.32	ICD-10-CM	Chronic kidney disease, stage 3b
	N18.4	ICD-10-CM	Chronic kidney disease, stage 4 (severe)
	N18.5	ICD-10-CM	Chronic kidney disease, stage 5
	N18.6	ICD-10-CM	End stage renal disease
	N18.9	ICD-10-CM	Chronic kidney disease, unspecified
Dyslipidaemia	E78.5	ICD-10-CM	Hyperlipidaemia, unspecified
GERD	530.11	ICD-9-CM	Reflux esophagitis
	530.81	ICD-9-CM	Oesophageal reflux
	787.1	ICD-9-CM	Heartburn
	K21	ICD-10-CM	Gastro-oesophageal reflux disease
	K21.0	ICD-10-CM	Gastro-oesophageal reflux disease with esophagitis
	K21.9	ICD-10-CM	Gastro-oesophageal reflux disease without esophagitis
	R12	ICD-10-CM	Heartburn
HFpEF	428.3	ICD-9-CM	Diastolic heart failure
	428.30	ICD-9-CM	Unspecified diastolic heart failure
	428.31	ICD-9-CM	Acute diastolic heart failure
	428.32	ICD-9-CM	Chronic diastolic heart failure
	428.33	ICD-9-CM	Acute on chronic diastolic heart failure
	I50.3	ICD-10-CM	Diastolic (congestive) heart failure
	I50.30	ICD-10-CM	Unspecified diastolic (congestive) heart failure
	I50.31	ICD-10-CM	Acute diastolic (congestive) heart failure
	I50.32	ICD-10-CM	Chronic diastolic (congestive) heart failure
	I50.33	ICD-10-CM	Acute on chronic diastolic (congestive) heart failure

Hypertension	401	ICD-9-CM	Essential hypertension
	401.1	ICD-9-CM	Benign essential hypertension
	401.9	ICD-9-CM	Unspecified essential hypertension
	I10	ICD-10-CM	Essential (primary) hypertension
Musculoskeletal pain	719.47	ICD-9-CM	Pain in joint involving ankle and foot
	723.1	ICD-9-CM	Cervicalgia
	724.3	ICD-9-CM	Sciatica
	724.5	ICD-9-CM	Backache, unspecified
	728.85	ICD-9-CM	Spasm of muscle
	M25.50	ICD-10-CM	Pain in unspecified joint
	M25.551	ICD-10-CM	Pain in right hip
	M25.571	ICD-10-CM	Pain in right ankle and joints of right foot
	M79.605	ICD-10-CM	Pain in left leg
	M79.609	ICD-10-CM	Pain in unspecified limb
	M79.659	ICD-10-CM	Pain in unspecified thigh
	M79.661	ICD-10-CM	Pain in right lower leg
	M79.676	ICD-10-CM	Pain in unspecified toe(s)
	338	ICD-9-CM	Pain, not elsewhere classified
	338.19	ICD-9-CM	Other acute pain
	338.2	ICD-9-CM	Chronic pain
	719.4	ICD-9-CM	Pain in joint
	723.8	ICD-9-CM	Other syndromes affecting cervical region
	724.2	ICD-9-CM	Lumbago
	M25.57	ICD-10-CM	Pain in ankle and joints of foot
	M25.579	ICD-10-CM	Pain in unspecified ankle and joints of unspecified foot
	M54.1	ICD-10-CM	Radiculopathy
	M54.32	ICD-10-CM	Sciatica, left side
	M54.8	ICD-10-CM	Other dorsalgia
	M62.831	ICD-10-CM	Muscle spasm of calf
	M62.838	ICD-10-CM	Other muscle spasm

	M79.6	ICD-10-CM	Pain in limb, hand, foot, fingers and toes
	M79.662	ICD-10-CM	Pain in left lower leg
	M79.674	ICD-10-CM	Pain in right toe(s)
	338.29	ICD-9-CM	Other chronic pain
	719.48	ICD-9-CM	Pain in joint involving other specified sites
	G89.1	ICD-10-CM	Acute pain, not elsewhere classified
	M25.552	ICD-10-CM	Pain in left hip
	M25.569	ICD-10-CM	Pain in unspecified knee
	M54.00	ICD-10-CM	Panniculitis affecting regions of neck and back, site unspecified
	M54.01	ICD-10-CM	Panniculitis affecting regions of neck and back, occipito-atlanto-axial region
	M54.03	ICD-10-CM	Panniculitis affecting regions of neck and back, cervicothoracic region
	M54.30	ICD-10-CM	Sciatica, unspecified side
	M54.41	ICD-10-CM	Lumbago with sciatica, right side
	M54.5	ICD-10-CM	Low back pain
	M54.81	ICD-10-CM	Occipital neuralgia
	M79.1	ICD-10-CM	Myalgia
	M79.10	ICD-10-CM	Myalgia, unspecified site
	M79.604	ICD-10-CM	Pain in right leg
	M79.651	ICD-10-CM	Pain in right thigh
	M79.652	ICD-10-CM	Pain in left thigh
	M79.66	ICD-10-CM	Pain in lower leg
	M79.673	ICD-10-CM	Pain in unspecified foot
	338.21	ICD-9-CM	Chronic pain due to trauma
	719.46	ICD-9-CM	Pain in joint involving lower leg
	719.49	ICD-9-CM	Pain in joint involving multiple sites
	724.8	ICD-9-CM	Other symptoms referable to back
	729.82	ICD-9-CM	Cramp of limb
	G89	ICD-10-CM	Pain, not elsewhere classified
	G89.2	ICD-10-CM	Chronic pain, not elsewhere classified
	M25.5	ICD-10-CM	Pain in joint

	M25.559	ICD-10-CM	Pain in unspecified hip
	M25.56	ICD-10-CM	Pain in knee
	M54.3	ICD-10-CM	Sciatica
	M54.31	ICD-10-CM	Sciatica, right side
	M54.4	ICD-10-CM	Lumbago with sciatica
	M54.6	ICD-10-CM	Pain in thoracic spine
	M62.83	ICD-10-CM	Muscle spasm
	M79.65	ICD-10-CM	Pain in thigh
	M79.671	ICD-10-CM	Pain in right foot
	M79.675	ICD-10-CM	Pain in left toe(s)
	338.1	ICD-9-CM	Acute pain
	719.40	ICD-9-CM	Pain in joint, site unspecified
	719.45	ICD-9-CM	Pain in joint involving pelvic region and thigh
	724.1	ICD-9-CM	Pain in thoracic spine
	780.96	ICD-9-CM	Generalized pain
	M25.55	ICD-10-CM	Pain in hip
	M25.572	ICD-10-CM	Pain in left ankle and joints of left foot
	M54.40	ICD-10-CM	Lumbago with sciatica, unspecified side
	M54.9	ICD-10-CM	Dorsalgia, unspecified
	M79.12	ICD-10-CM	Myalgia of auxiliary muscles, head and neck
	M79.18	ICD-10-CM	Myalgia, other site
	M79.60	ICD-10-CM	Pain in limb, unspecified
	M79.669	ICD-10-CM	Pain in unspecified lower leg
	729.5	ICD-9-CM	Pain in limb
	G89.29	ICD-10-CM	Other chronic pain
	M25.561	ICD-10-CM	Pain in right knee
	M25.562	ICD-10-CM	Pain in left knee
	M54	ICD-10-CM	Dorsalgia
	M54.2	ICD-10-CM	Cervicalgia
	M54.42	ICD-10-CM	Lumbago with sciatica, left side

	M54.89	ICD-10-CM	Other dorsalgia
	M62.830	ICD-10-CM	Muscle spasm of back
	M79.606	ICD-10-CM	Pain in leg, unspecified
	M79.67	ICD-10-CM	Pain in foot and toes
	M79.672	ICD-10-CM	Pain in left foot
	R52	ICD-10-CM	Pain, unspecified
OSA	327.23	ICD-9-CM	Obstructive sleep apnoea (adult)(paediatric)
	G47.33	ICD-10-CM	Obstructive sleep apnoea (adult) (paediatric)
OA of the knee	715.16	ICD-9-CM	Osteoarthrosis, localized, primary, involving lower leg
	715.36	ICD-9-CM	Osteoarthrosis, localized, not specified whether primary or secondary, involving lower leg
	715.96	ICD-9-CM	Osteoarthrosis, unspecified whether generalized or localized, involving lower leg
	M17	ICD-10-CM	Osteoarthritis of knee
	M17.0	ICD-10-CM	Bilateral primary osteoarthritis of knee
	M17.1	ICD-10-CM	Unilateral primary osteoarthritis of knee
	M17.10	ICD-10-CM	Unilateral primary osteoarthritis, unspecified knee
	M17.11	ICD-10-CM	Unilateral primary osteoarthritis, right knee
	M17.12	ICD-10-CM	Unilateral primary osteoarthritis, left knee
	M17.9	ICD-10-CM	Osteoarthritis of knee, unspecified
Prediabetes	790.2	ICD-9-CM	Abnormal glucose
	790.21	ICD-9-CM	Impaired fasting glucose
	790.22	ICD-9-CM	Impaired glucose tolerance test (oral)
	790.29	ICD-9-CM	Other abnormal glucose
	R73.0	ICD-10-CM	Abnormal glucose
	R73.01	ICD-10-CM	Impaired fasting glucose
	R73.02	ICD-10-CM	Impaired glucose tolerance (oral)
	R73.03	ICD-10-CM	Prediabetes
	R73.09	ICD-10-CM	Other abnormal glucose
Psoriasis	696.0	ICD-9-CM	Psoriatic arthropathy
	696.1	ICD-9-CM	Other psoriasis and similar disorders
	696.8	ICD-9-CM	Other psoriasis and similar disorders

	L40	ICD-10-CM	Psoriasis
	L40.0	ICD-10-CM	Psoriasis vulgaris
	L40.1	ICD-10-CM	Generalized pustular psoriasis
	L40.4	ICD-10-CM	Guttate psoriasis
	L40.5	ICD-10-CM	Arthropathic psoriasis
	L40.50	ICD-10-CM	Arthropathic psoriasis, unspecified
	L40.51	ICD-10-CM	Distal interphalangeal psoriatic arthropathy
	L40.52	ICD-10-CM	Psoriatic arthritis mutilans
	L40.53	ICD-10-CM	Psoriatic spondylitis
	L40.54	ICD-10-CM	Psoriatic juvenile arthropathy
	L40.59	ICD-10-CM	Other psoriatic arthropathy
	L40.8	ICD-10-CM	Other psoriasis
	L40.9	ICD-10-CM	Psoriasis, unspecified
T2D	250.00	ICD-9-CM	Diabetes mellitus without mention of complication, type II or unspecified type, not stated as uncontrolled
	250.02	ICD-9-CM	Diabetes mellitus without mention of complication, type II or unspecified type, uncontrolled
	250.10	ICD-9-CM	Diabetes with ketoacidosis, type II or unspecified type, not stated as uncontrolled
	250.12	ICD-9-CM	Diabetes with ketoacidosis, type II or unspecified type, uncontrolled
	250.20	ICD-9-CM	Diabetes with hyperosmolarity, type II or unspecified type, not stated as uncontrolled
	250.22	ICD-9-CM	Diabetes with hyperosmolarity, type II or unspecified type, uncontrolled
	250.30	ICD-9-CM	Diabetes with other coma, type II or unspecified type, not stated as uncontrolled
	250.32	ICD-9-CM	Diabetes with other coma, type II or unspecified type, uncontrolled
	250.40	ICD-9-CM	Diabetes with renal manifestations, type II or unspecified type, not stated as uncontrolled
	250.42	ICD-9-CM	Diabetes with renal manifestations, type II or unspecified type, uncontrolled
	250.50	ICD-9-CM	Diabetes with ophthalmic manifestations, type II or unspecified type, not stated as uncontrolled
	250.52	ICD-9-CM	Diabetes with ophthalmic manifestations, type II or unspecified type, uncontrolled
	250.60	ICD-9-CM	Diabetes with neurological manifestations, type II or unspecified type, not stated as uncontrolled
	250.62	ICD-9-CM	Diabetes with neurological manifestations, type II or unspecified type, uncontrolled

	250.70	ICD-9-CM	Diabetes with peripheral circulatory disorders, type II or unspecified type, not stated as uncontrolled
	250.72	ICD-9-CM	Diabetes with peripheral circulatory disorders, type II or unspecified type, uncontrolled
	250.80	ICD-9-CM	Diabetes with other specified manifestations, type II or unspecified type, not stated as uncontrolled
	250.82	ICD-9-CM	Diabetes with other specified manifestations, type II or unspecified type, uncontrolled
	250.90	ICD-9-CM	Diabetes with unspecified complication, type II or unspecified type, not stated as uncontrolled
	250.92	ICD-9-CM	Diabetes with unspecified complication, type II or unspecified type, uncontrolled
	E11	ICD-10-CM	Type 2 diabetes mellitus
	E11.0	ICD-10-CM	Type 2 diabetes mellitus with hyperosmolarity
	E11.00	ICD-10-CM	Type 2 diabetes mellitus with hyperosmolarity without nonketotic hyperglycaemic-hyperosmolar coma
	E11.01	ICD-10-CM	Type 2 diabetes mellitus with hyperosmolarity with coma
	E11.1	ICD-10-CM	Type 2 diabetes mellitus with ketoacidosis
	E11.10	ICD-10-CM	Type 2 diabetes mellitus with ketoacidosis without coma
	E11.11	ICD-10-CM	Type 2 diabetes mellitus with ketoacidosis with coma
	E11.2	ICD-10-CM	Type 2 diabetes mellitus with kidney complications
	E11.21	ICD-10-CM	Type 2 diabetes mellitus with diabetic nephropathy
	E11.22	ICD-10-CM	Type 2 diabetes mellitus with diabetic chronic kidney disease
	E11.29	ICD-10-CM	Type 2 diabetes mellitus with other diabetic kidney complication
	E11.3	ICD-10-CM	Type 2 diabetes mellitus with ophthalmic complications
	E11.31	ICD-10-CM	Type 2 diabetes mellitus with unspecified diabetic retinopathy
	E11.311	ICD-10-CM	Type 2 diabetes mellitus with unspecified diabetic retinopathy with macular oedema
	E11.319	ICD-10-CM	Type 2 diabetes mellitus with unspecified diabetic retinopathy without macular oedema
	E11.32	ICD-10-CM	Type 2 diabetes mellitus with mild nonproliferative diabetic retinopathy
	E11.321	ICD-10-CM	Type 2 diabetes mellitus with mild nonproliferative diabetic retinopathy with macular oedema
	E11.3211	ICD-10-CM	Type 2 diabetes mellitus with mild nonproliferative diabetic retinopathy with macular oedema, right eye
	E11.3212	ICD-10-CM	Type 2 diabetes mellitus with mild nonproliferative diabetic retinopathy with macular oedema, left eye

	E11.3213	ICD-10-CM	Type 2 diabetes mellitus with mild nonproliferative diabetic retinopathy with macular oedema, bilateral
	E11.3219	ICD-10-CM	Type 2 diabetes mellitus with mild nonproliferative diabetic retinopathy with macular oedema, unspecified eye
	E11.329	ICD-10-CM	Type 2 diabetes mellitus with mild nonproliferative diabetic retinopathy without macular oedema
	E11.3291	ICD-10-CM	Type 2 diabetes mellitus with mild nonproliferative diabetic retinopathy without macular oedema, right eye
	E11.3292	ICD-10-CM	Type 2 diabetes mellitus with mild nonproliferative diabetic retinopathy without macular oedema, left eye
	E11.3293	ICD-10-CM	Type 2 diabetes mellitus with mild nonproliferative diabetic retinopathy without macular oedema, bilateral
	E11.3299	ICD-10-CM	Type 2 diabetes mellitus with mild nonproliferative diabetic retinopathy without macular oedema, unspecified eye
	E11.33	ICD-10-CM	Type 2 diabetes mellitus with moderate nonproliferative diabetic retinopathy
	E11.331	ICD-10-CM	Type 2 diabetes mellitus with moderate nonproliferative diabetic retinopathy with macular oedema
	E11.3311	ICD-10-CM	Type 2 diabetes mellitus with moderate nonproliferative diabetic retinopathy with macular oedema, right eye
	E11.3312	ICD-10-CM	Type 2 diabetes mellitus with moderate nonproliferative diabetic retinopathy with macular oedema, left eye
	E11.3313	ICD-10-CM	Type 2 diabetes mellitus with moderate nonproliferative diabetic retinopathy with macular oedema, bilateral
	E11.3319	ICD-10-CM	Type 2 diabetes mellitus with moderate nonproliferative diabetic retinopathy with macular oedema, unspecified eye
	E11.339	ICD-10-CM	Type 2 diabetes mellitus with moderate nonproliferative diabetic retinopathy without macular oedema
	E11.3391	ICD-10-CM	Type 2 diabetes mellitus with moderate nonproliferative diabetic retinopathy without macular oedema, right eye

	E11.3392	ICD-10-CM	Type 2 diabetes mellitus with moderate nonproliferative diabetic retinopathy without macular oedema, left eye
	E11.3393	ICD-10-CM	Type 2 diabetes mellitus with moderate nonproliferative diabetic retinopathy without macular oedema, bilateral
	E11.3399	ICD-10-CM	Type 2 diabetes mellitus with moderate nonproliferative diabetic retinopathy without macular oedema, unspecified eye
	E11.34	ICD-10-CM	Type 2 diabetes mellitus with severe nonproliferative diabetic retinopathy
	E11.341	ICD-10-CM	Type 2 diabetes mellitus with severe nonproliferative diabetic retinopathy with macular oedema
	E11.3411	ICD-10-CM	Type 2 diabetes mellitus with severe nonproliferative diabetic retinopathy with macular oedema, right eye
	E11.3412	ICD-10-CM	Type 2 diabetes mellitus with severe nonproliferative diabetic retinopathy with macular oedema, left eye
	E11.3413	ICD-10-CM	Type 2 diabetes mellitus with severe nonproliferative diabetic retinopathy with macular oedema, bilateral
	E11.3419	ICD-10-CM	Type 2 diabetes mellitus with severe nonproliferative diabetic retinopathy with macular oedema, unspecified eye
	E11.349	ICD-10-CM	Type 2 diabetes mellitus with severe nonproliferative diabetic retinopathy without macular oedema
	E11.3491	ICD-10-CM	Type 2 diabetes mellitus with severe nonproliferative diabetic retinopathy without macular oedema, right eye
	E11.3492	ICD-10-CM	Type 2 diabetes mellitus with severe nonproliferative diabetic retinopathy without macular oedema, left eye
	E11.3493	ICD-10-CM	Type 2 diabetes mellitus with severe nonproliferative diabetic retinopathy without macular oedema, bilateral
	E11.3499	ICD-10-CM	Type 2 diabetes mellitus with severe nonproliferative diabetic retinopathy without macular oedema, unspecified eye
	E11.35	ICD-10-CM	Type 2 diabetes mellitus with proliferative diabetic retinopathy
	E11.351	ICD-10-CM	Type 2 diabetes mellitus with proliferative diabetic retinopathy with macular oedema
	E11.3511	ICD-10-CM	Type 2 diabetes mellitus with proliferative diabetic retinopathy with macular oedema, right eye
	E11.3512	ICD-10-CM	Type 2 diabetes mellitus with proliferative diabetic retinopathy with macular oedema, left eye

	E11.3513	ICD-10-CM	Type 2 diabetes mellitus with proliferative diabetic retinopathy with macular oedema, bilateral
	E11.3519	ICD-10-CM	Type 2 diabetes mellitus with proliferative diabetic retinopathy with macular oedema, unspecified eye
	E11.352	ICD-10-CM	Type 2 diabetes mellitus with proliferative diabetic retinopathy with traction retinal detachment involving the macula
	E11.3521	ICD-10-CM	Type 2 diabetes mellitus with proliferative diabetic retinopathy with traction retinal detachment involving the macula, right eye
	E11.3522	ICD-10-CM	Type 2 diabetes mellitus with proliferative diabetic retinopathy with traction retinal detachment involving the macula, left eye
	E11.3523	ICD-10-CM	Type 2 diabetes mellitus with proliferative diabetic retinopathy with traction retinal detachment involving the macula, bilateral
	E11.3529	ICD-10-CM	Type 2 diabetes mellitus with proliferative diabetic retinopathy with traction retinal detachment involving the macula, unspecified eye
	E11.353	ICD-10-CM	Type 2 diabetes mellitus with proliferative diabetic retinopathy with traction retinal detachment not involving the macula
	E11.3531	ICD-10-CM	Type 2 diabetes mellitus with proliferative diabetic retinopathy with traction retinal detachment not involving the macula, right eye
	E11.3532	ICD-10-CM	Type 2 diabetes mellitus with proliferative diabetic retinopathy with traction retinal detachment not involving the macula, left eye
	E11.3533	ICD-10-CM	Type 2 diabetes mellitus with proliferative diabetic retinopathy with traction retinal detachment not involving the macula, bilateral
	E11.3539	ICD-10-CM	Type 2 diabetes mellitus with proliferative diabetic retinopathy with traction retinal detachment not involving the macula, unspecified eye
	E11.354	ICD-10-CM	Type 2 diabetes mellitus with proliferative diabetic retinopathy with combined traction retinal detachment and rhegmatogenous retinal detachment
	E11.3541	ICD-10-CM	Type 2 diabetes mellitus with proliferative diabetic retinopathy with combined traction retinal detachment and rhegmatogenous retinal detachment, right eye
	E11.3542	ICD-10-CM	Type 2 diabetes mellitus with proliferative diabetic retinopathy with combined traction retinal detachment and rhegmatogenous retinal detachment, left eye

	E11.3543	ICD-10-CM	Type 2 diabetes mellitus with proliferative diabetic retinopathy with combined traction retinal detachment and rhegmatogenous retinal detachment, bilateral
	E11.3549	ICD-10-CM	Type 2 diabetes mellitus with proliferative diabetic retinopathy with combined traction retinal detachment and rhegmatogenous retinal detachment, unspecified eye
	E11.355	ICD-10-CM	Type 2 diabetes mellitus with stable proliferative diabetic retinopathy
	E11.3551	ICD-10-CM	Type 2 diabetes mellitus with stable proliferative diabetic retinopathy, right eye
	E11.3552	ICD-10-CM	Type 2 diabetes mellitus with stable proliferative diabetic retinopathy, left eye
	E11.3553	ICD-10-CM	Type 2 diabetes mellitus with stable proliferative diabetic retinopathy, bilateral
	E11.3559	ICD-10-CM	Type 2 diabetes mellitus with stable proliferative diabetic retinopathy, unspecified eye
	E11.359	ICD-10-CM	Type 2 diabetes mellitus with proliferative diabetic retinopathy without macular oedema
	E11.3591	ICD-10-CM	Type 2 diabetes mellitus with proliferative diabetic retinopathy without macular oedema, right eye
	E11.3592	ICD-10-CM	Type 2 diabetes mellitus with proliferative diabetic retinopathy without macular oedema, left eye
	E11.3593	ICD-10-CM	Type 2 diabetes mellitus with proliferative diabetic retinopathy without macular oedema, bilateral
	E11.3599	ICD-10-CM	Type 2 diabetes mellitus with proliferative diabetic retinopathy without macular oedema, unspecified eye
	E11.36	ICD-10-CM	Type 2 diabetes mellitus with diabetic cataract
	E11.37	ICD-10-CM	Type 2 diabetes mellitus with diabetic macular oedema, resolved following treatment
	E11.37X1	ICD-10-CM	Type 2 diabetes mellitus with diabetic macular oedema, resolved following treatment, right eye
	E11.37X2	ICD-10-CM	Type 2 diabetes mellitus with diabetic macular oedema, resolved following treatment, left eye
	E11.37X3	ICD-10-CM	Type 2 diabetes mellitus with diabetic macular oedema, resolved following treatment, bilateral
	E11.37X9	ICD-10-CM	Type 2 diabetes mellitus with diabetic macular oedema, resolved following treatment, unspecified eye
	E11.39	ICD-10-CM	Type 2 diabetes mellitus with other diabetic ophthalmic complication
	E11.4	ICD-10-CM	Type 2 diabetes mellitus with neurological complications
	E11.40	ICD-10-CM	Type 2 diabetes mellitus with diabetic neuropathy, unspecified
	E11.41	ICD-10-CM	Type 2 diabetes mellitus with diabetic mononeuropathy
	E11.42	ICD-10-CM	Type 2 diabetes mellitus with diabetic polyneuropathy

	E11.43	ICD-10-CM	Type 2 diabetes mellitus with diabetic autonomic (poly)neuropathy
	E11.44	ICD-10-CM	Type 2 diabetes mellitus with diabetic amyotrophy
	E11.49	ICD-10-CM	Type 2 diabetes mellitus with other diabetic neurological complication
	E11.5	ICD-10-CM	Type 2 diabetes mellitus with circulatory complications
	E11.51	ICD-10-CM	Type 2 diabetes mellitus with diabetic peripheral angiopathy without gangrene
	E11.52	ICD-10-CM	Type 2 diabetes mellitus with diabetic peripheral angiopathy with gangrene
	E11.59	ICD-10-CM	Type 2 diabetes mellitus with other circulatory complications
	E11.6	ICD-10-CM	Type 2 diabetes mellitus with other specified complications
	E11.61	ICD-10-CM	Type 2 diabetes mellitus with diabetic arthropathy
	E11.610	ICD-10-CM	Type 2 diabetes mellitus with diabetic neuropathic arthropathy
	E11.618	ICD-10-CM	Type 2 diabetes mellitus with other diabetic arthropathy
	E11.62	ICD-10-CM	Type 2 diabetes mellitus with skin complications
	E11.620	ICD-10-CM	Type 2 diabetes mellitus with diabetic dermatitis
	E11.621	ICD-10-CM	Type 2 diabetes mellitus with foot ulcer
	E11.622	ICD-10-CM	Type 2 diabetes mellitus with other skin ulcer
	E11.628	ICD-10-CM	Type 2 diabetes mellitus with other skin complications
	E11.63	ICD-10-CM	Type 2 diabetes mellitus with oral complications
	E11.630	ICD-10-CM	Type 2 diabetes mellitus with periodontal disease
	E11.638	ICD-10-CM	Type 2 diabetes mellitus with other oral complications
	E11.64	ICD-10-CM	Type 2 diabetes mellitus with hypoglycaemia
	E11.641	ICD-10-CM	Type 2 diabetes mellitus with hypoglycaemia with coma
	E11.649	ICD-10-CM	Type 2 diabetes mellitus with hypoglycaemia without coma
	E11.65	ICD-10-CM	Type 2 diabetes mellitus with hyperglycaemia
	E11.69	ICD-10-CM	Type 2 diabetes mellitus with other specified complication
	E11.8	ICD-10-CM	Type 2 diabetes mellitus with unspecified complications
	E11.9	ICD-10-CM	Type 2 diabetes mellitus without complications
Urinary incontinence	625.6	ICD-9-CM	Stress incontinence, female
	788.3	ICD-9-CM	Urinary incontinence
	788.31	ICD-9-CM	Urge incontinence
	788.33	ICD-9-CM	Mixed incontinence (male) (female)

	788.35	ICD-9-CM	Post-void dribbling
	N39.3	ICD-10-CM	Stress incontinence (female) (male)
	N39.4	ICD-10-CM	Other specified urinary incontinence
	N39.41	ICD-10-CM	Urge incontinence
	N39.43	ICD-10-CM	Post-void dribbling
	N39.46	ICD-10-CM	Mixed incontinence
	N39.492	ICD-10-CM	Postural (urinary) incontinence

ASCVD atherosclerotic cardiovascular disease, *CKD* chronic kidney disease, *GERD* gastro-oesophageal reflux disease, *HFpEF* heart failure with preserved ejection fraction, *ICD-9/10-CM* International Classification of Diseases Version 9/10 clinical modification, *OA* osteoarthritis, *ORC* obesity-related complication, *OSA* obstructive sleep apnoea; *PCOS* polycystic ovary syndrome, *T2D* type 2 diabetes.

Table S2 Attrition table.

Initial sample: 16,981,714 patients with ≥1 BMI value ≥30.0 kg/m^2 and <70.0 kg/m^2 in AEMR between 1 January 2007 through 31 March 2017*

n (%)	Obesity class I	Obesity class II	Obesity class III
Individuals from initial sample with BMI ≥30.0–<35.0 kg/m^2	10 103 478 (100.0)		
Individuals from initial sample with BMI ≥35.0–<40.0 kg/m^2		3 894 054 (100.0)	
Individuals from initial sample with BMI ≥40.0–<70.0 kg/m^2			2 984 182 (100.0)
Linkage to PharMetrics Plus during the study period	2 615 421 (25.9)	1 006 428 (25.8)	764 360 (25.6)
Individuals with continuous enrollment in PharMetrics Plus 1-year pre-index date	658 010 (6.5)	243 378 (6.2)	177 013 (5.9)
Continuous enrolment in PharMetrics Plus ≥3 years post-index date	312 666 (3.1)	114 107 (2.9)	81 064 (2.7)
≥18 years at index date	311 082 (3.1)	113 481 (2.9)	80 501 (2.7)
Newly diagnosed with obesity class I, II or III**	278 328 (2.8)	102 319 (2.6)	64 148 (2.1)
Individuals without ≥1 pregnancy diagnosis in the 1-year baseline period	269 414 (2.7)	100 324 (2.6)	62 924 (2.1)
Individuals without ≥1 cancer diagnosis (except non-melanoma skin cancer) during all available pre-index period with patient continuously enrolled	159 584 (1.6)	60 534 (1.6)	39 854 (1.3)
Individuals without incomplete data or data quality issues (total; below steps applied sequentially)	138 340 (1.4)	51 493 (1.3)	33 371 (1.1)

Without Medicare Cost coverage or SCHIP	157 083 (1.6)	59 631 (1.5)	39 418 (1.3)
Without invalid/missing year of birth, sex, region or health plan enrollment dates	151 601 (1.5)	57 593 (1.5)	37 924 (1.3)
Without duplicated linkage (1:N or N:1 linkage across databases)	140 447 (1.4)	52 576 (1.4)	34 825 (1.2)
Without >20% change in BMI between consecutive BMI measurements within 30 days of each other, or >20% average monthly change in BMI between consecutive BMI measurements that are >1 month apart anytime during pre-index and available post-index	138 472 (1.4)	51 543 (1.3)	33 410 (1.1)
Without incomplete claims information	138 340 (1.4)	51 493 (1.3)	33 371 (1.1)
Patients with continuous enrolment in PharMetrics Plus ≥8 years following their index date	17 892 (0.2)	6550 (0.2)	4141 (0.1)

*The first BMI value (recorded or calculated [using the weight value]) was termed the index date.

**Without ≥1 BMI value ≥30.0kg/m^2 or specific class I, class II or class III obesity diagnosis codes or non-specific class I or class II obesity diagnosis codes for cohort 1 (obesity class I); without ≥1 BMI value ≥35.0kg/m^2 or specific class II or class III obesity diagnosis codes for cohort 2 (obesity class II); or without ≥1 BMI value ≥40.0kg/m^2 or specific class III obesity diagnosis codes for cohort 3 (obesity class III) in the 1-year baseline period, respectively

Table S3 Baseline characteristics of individuals with obesity, by specific ORC.

Baseline characteristics	No ORCs[a] *n* = 12 686	With T2D *n* = 2611	With established CVD[a] *n* = 2091	With ASCVD[b] *n* = 1662	With HF *n* = 197	With CKD *n* = 216	With OA of knee *n* = 873	≥2 ORCs[a] *n* = 8655	≥3 ORCs[a] *n* = 4475
**Age, years, mean (SD)**	43.1 (10.6)	51.7 (8.9)	53.2 (9.1)	53.7 (8.9)	53.1 (9.6)	54.0 (11.5)	53.1 (8.4)	50.1 (9.3)	51.7 (8.9)
**Women, n (%)**	5737 (45.2)	1156 (44.3)	714 (34.1)	526 (31.6)	64 (32.5)	89 (41.2)	457 (52.3)	3949 (45.6)	2004 (44.8)
**CCI score, mean (SD)**	0.0 (0.2)	1.5 (0.9)	1.2 (1.2)	1.3 (1.3)	2.3 (1.4)	3.0 (1.4)	0.6 (0.9)	0.7 (0.9)	1.0 (1.1)
**Region, n (%)**									
Northeast	4426 (34.9)	841 (32.2)	555 (26.5)	437 (26.3)	42 (21.3)	59 (27.3)	310 (35.5)	2926 (33.8)	1451 (32.4)
Midwest	4157 (32.8)	664 (25.4)	444 (21.2)	337 (20.3)	37 (18.8)	53 (24.5)	232 (26.6)	2151 (24.9)	1053 (23.5)
South	3319 (26.2)	960 (36.8)	974 (46.6)	796 (47.9)	109 (55.3)	84 (38.9)	295 (33.8)	3124 (36.1)	1746 (39.0)
West	784 (6.2)	146 (5.6)	118 (5.6)	92 (5.5)	9 (4.6)	20 (9.3)	36 (4.1)	454 (5.2)	225 (5.0)
**Race/ethnicity, n (%)**									
White	8393 (66.2)	1727 (66.1)	1471 (70.3)	1184 (71.2)	121 (61.4)	135 (62.5)	602 (69.0)	5964 (68.9)	3109 (69.5)
African American	462 (3.6)	165 (6.3)	79 (3.8)	59 (3.5)	15 (7.6)	16 (7.4)	35 (4.0)	390 (4.5)	196 (4.4)
Asian	84 (0.7)	20 (0.8)	6 (0.3)	6 (0.4)	0 (0)	3 (1.4)	0 (0)	46 (0.5)	19 (0.4)
Hispanic	88 (0.7)	22 (0.8)	13 (0.6)	12 (0.7)	2 (1.0)	1 (0.5)	10 (1.1)	51 (0.6)	22 (0.5)

Unknown	3659 (28.8)	677 (25.9)	522 (25.0)	401 (24.1)	59 (29.9)	61 (28.2)	226 (25.9)	2204 (25.5)	1129 (25.2)
**Payer type, n (%)**									
Commercial	6979 (55.0)	1301 (49.8)	1120 (53.6)	896 (53.9)	106 (53.8)	115 (53.2)	450 (51.5)	4492 (51.9)	2288 (51.1)
Medicaid	43 (0.3)	6(0.2)	2 (0.1)	2 (0.1)	0 (0)	0 (0)	0 (0)	20 (0.2)	3 (0.1)
Medicare Risk	39 (0.3)	74 (2.8)	86 (4.1)	67 (4.0)	13 (6.6)	15 (6.9)	25 (2.9)	186 (2.1)	134 (3.0)
Self-insured	5619 (44.3)	1230 (47.1)	883 (42.2)	697 (41.9)	78 (39.6)	86 (39.8)	398 (45.6)	3957 (45.7)	2050 (45.8)
Unknown	6 (<0.1)	0 (0)	0 (0)	0 (0)	0 (0)	0 (0)	0 (0)	0 (0)	0 (0)

ORCs of interest were obstructive sleep apnoea, HF, urinary incontinence, OA of the knee, T2D, prediabetes, asthma, psoriasis, gastro-oesophageal reflux disease, hypertension, dyslipidaemia, musculoskeletal pain, ASCVD and CKD.

CCI Charlson comorbidity index, *CKD* chronic kidney disease, *CVD* cardiovascular disease, *HF* heart failure, *OA* osteoarthritis, *ORC* obesity-related complication, *T2D* type 2 diabetes, *ASCVD* atherosclerotic cardiovascular disease.

[a]Established CVD included ASCVD, HF, cardiomyopathies, deep vein thrombosis and pulmonary embolism, cardiac arrest, atrial fibrillation and flutter, and atherosclerosis.

[b]ASCVD included cerebrovascular disease, ischaemic heart disease and peripheral artery disease.

Table S4 Adjusted mean annual total all-cause per-person healthcare costs at baseline and annually among individuals with obesity, by specific ORC.

Costs, 2019 US$	Year 1	Year 2	Year 3	Year 4	Year 5	Year 6	Year 7	Year 8
With T2D	14 744	13 159	14 822	16 087	18 059	18 987	20 631	21 798
Without T2D	7260	6228	6607	6891	7219	7526	8070	9195
With established CVD	17 534	14 668	14 939	15 861	16 941	17 270	18 965	20 380
Without established CVD	7210	6255	6753	7074	7496	7855	8416	9528
With HF	26 424	21 450	23 156	22 565	26 165	23 991	29 531	27 535
Without HF	7822	6762	7240	7614	8050	8425	9026	10 185
With CKD	27 339	24 109	31 820	29 735	41 962	47 323	44 204	48 888
Without CKD	7825	6749	7189	7573	7955	8282	8939	10 064
With OA of the knee	17 472	14 668	16 381	14 354	16 595	14 610	16 872	18 083
Without OA of the knee	7656	6620	7073	7506	7911	8331	8919	10 055
With ≥2 ORCs[a]	N/A	N/A	N/A	N/A	N/A	N/A	N/A	N/A
With ≥3 ORCs[a]	15 585	13 117	14 636	15 018	16 655	17 192	19 057	20 349
With high CV risk[b]	13 222	11 231	12 360	13 466	14 515	15 393	16 585	17 899

ASCVD atherosclerotic cardiovascular disease, *CKD* chronic kidney disease, *CVD* cardiovascular disease, *HF* heart failure, *N/A* not applicable, *OA* osteoarthritis, *ORC* obesity-related complication, *T2D* type 2 diabetes.

[a]ORCs were obstructive sleep apnoea, HF, urinary incontinence, OA of the knee, T2D, prediabetes, asthma, psoriasis, gastro-oesophageal reflux disease, hypertension, dyslipidaemia, musculoskeletal pain, ASCVD and CKD.

[b]High CV risk: ≥2 risk factors out of hypertension, dyslipidaemia and T2D/prediabetes.

Table S5 Observed mean total all-cause per-person healthcare costs at baseline and annually among individuals with obesity, stratified by specific ORC.

Costs per person per year, 2019 US$	Baseline year	Year 1	Year 2	Year 3	Year 4	Year 5	Year 6	Year 7	Year 8
With T2D	10 929	14 757	12 980	14 682	15 689	17 481	18 460	20 065	21 105
Without T2D	4595	7232	6213	6627	6927	7253	7571	8157	9246
With established CVD	18 653	17 203	14 407	14 604	15 507	16 758	16 993	18 718	20 120
Without established CVD	4109	7186	6233	6792	7113	7511	7901	8497	9556
With HF	34 126	26 707	21 138	22 762	22 781	25 687	23 622	28 643	26 609
Without HF	4972	7789	6732	7256	7623	8066	8461	9110	10 216
With CKD	28 411	24 694	22 461	26 516	26 445	36 342	36 472	37 433	38 349
Without CKD	4996	7791	6712	7217	7584	7973	8353	9030	10 116
With OA of the knee	14 161	16 952	14 293	16 406	14 336	16 560	14 577	16 953	18 100
Without OA of the knee	4890	7635	6596	7078	7519	7924	8376	9002	10 084
With ≥2 ORCs[a]	10 813	12 618	10 732	11 946	12 552	13 351	13 693	14 925	16 050
With ≥3 ORCs[a]	14 290	15 571	13 043	14 544	14 949	16 575	17 080	18 915	20 078
With high CV risk[b]	10 831	13 340	11 196	12 304	13 312	14 341	15 235	16 403	17 711

ASCVD atherosclerotic cardiovascular disease, *CKD* chronic kidney disease, *CVD* cardiovascular disease, *HF* heart failure, *OA* osteoarthritis, *ORC* obesity-related complication, *T2D* type 2 diabetes.

[a]ORCs were obstructive sleep apnoea, HF, urinary incontinence, OA of the knee, T2D, prediabetes, asthma, psoriasis, gastro-oesophageal reflux disease, hypertension, dyslipidaemia, musculoskeletal pain, ASCVD and CKD.

[b]High CV risk: ≥2 risk factors out of hypertension, dyslipidaemia and T2D/prediabetes.

Table S6 Adjusted mean total cost per person per year of follow-up, stratified by obesity class.

**Costs, 2019 US$**		**Year 1**	**Year 2**	**Year 3**	**Year 4**	**Year 5**	**Year 6**	**Year 7**	**Year 8**
**T2D**	**Class I (*n* = 1213)**	15 115	13 018	15 000	14 944	17 922	17 365	19 492	20 047
	**Class II (*n* = 728)**	14 391	13 361	14 703	17 435	18 868	22 325	21 155	23 558
	**Class III (*n* = 670)**	14 456	13 194	14 627	16 691	17 429	18 296	22 124	23 054
**Established CVD**	**Class I (*n* = 1264)**	17 126	14 427	14 390	13 938	16 161	16 388	18 113	19 562
	**Class II (*n* = 516)**	17 921	15 054	15 544	18 755	17 261	19 137	19 802	21 836
	**Class III (*n* = 311)**	18 552	15 010	16 165	18 874	19 582	17 761	21 037	21 288
**HF**	**Class I (*n* = 100)**	26 331	24 409	19 097	13 950	22 341	20 738	31 547	27 574
	**Class II (*n* = 48)**	27 413	17 300	24 747	20 387	26 230	24 485	22 927	22 583
	**Class III (*n* = 49)**	25 648	19 475	29 881	42 280	33 908	30 147	31 886	32 306
**CKD**	**Class I (*n* = 144)**	29 176	19 994	29 932	20 640	37 894	40 302	42 597	55 447
	**Class II (*n* = 39)**	21 150	40 950	36 189	52 600	55 284	67 838	52 343	34 239
	**Class III (*n* = 33)**	26 639	22 165	34 899	42 400	43 968	53 714	41 598	37 580
**OA of the knee**	**Class I (*n* = 412)**	17 089	13 824	18 260	14 774	16 192	14 805	17 225	16 433
	**Class II (*n* = 259)**	16 945	15 753	13 261	12 388	15 522	12 314	17 628	18 212
	**Class III (*n* = 202)**	18 929	14 996	16 549	16 021	18 792	17 155	15 183	21 284
**High CV risk[a]**	**Class I (*n* = 2487)**	13 049	10 679	11 431	12 230	13 850	14 461	15 615	16 638
	**Class II (*n* = 1287)**	13 272	11 785	13 278	14 634	15 111	16 786	17 174	18 725

	**Class III (*n* = 917)**	13 623	11 950	13 593	15 179	15 485	15 969	18 387	20 160
**≥3 ORCs[b]**	**Class I (*n* = 2387)**	15 774	12 935	14 623	14 250	16 463	16 632	18 798	19 610
	**Class II (*n* = 1179)**	15 566	13 366	14 451	15 624	16 643	18 204	19 224	21 093
	**Class III (*n* = 909)**	15 115	13 272	14 911	16 251	17 173	17 352	19 519	21 325

ASCVD atherosclerotic cardiovascular disease, *CKD* chronic kidney disease, *CVD* cardiovascular disease, *HF* heart failure, *OA* osteoarthritis, *ORC* obesity-related complication, *T2D* type 2 diabetes.

[a]High CV risk: ≥2 risk factors out of hypertension, dyslipidaemia and T2D/prediabetes.

[b]ORCs were obstructive sleep apnoea, HF, urinary incontinence, OA of the knee, T2D, prediabetes, asthma, psoriasis, gastro-oesophageal reflux disease, hypertension, dyslipidaemia, musculoskeletal pain, ASCVD and CKD.

Table S7 Observed mean total all-cause healthcare cost per person per year of follow-up, stratified by obesity class.

Cost per person per year, 2019 US$		Year 1	Year 2	Year 3	Year 4	Year 5	Year 6	Year 7	Year 8
**T2D**	**Class I (*n* = 1213)**	15 242	12 904	15 036	14 770	17 438	16 955	18 945	19 416
	**Class II (*n* = 728)**	14 288	12 921	14 272	16 615	17 721	21 786	20 452	22 591
	**Class III (*n* = 670)**	14 388	13 182	14 488	16 347	17 298	17 569	21 674	22 549
**Established CVD**	**Class I (*n* = 1264)**	16 484	14 027	14 027	13 671	15 881	16 038	17 589	19 117
	**Class II (*n* = 516)**	17 925	14 674	14 840	17 709	17 055	19 006	19 901	21 708
	**Class III (*n* = 311)**	18 929	15 512	16 560	19 312	19 827	17 535	21 342	21 563
**HF**	**Class I (*n* = 100)**	26 288	23 636	19 079	14 681	21 564	19 515	29 125	26 253
	**Class II (*n* = 48)**	29 320	17 050	23 747	21 264	25 624	25 744	23 532	22 226
	**Class III (*n* = 49)**	25 002	20 047	29 313	40 795	34 163	29 925	32 667	31 629
**CKD**	**Class I (*n* = 144)**	25 807	18 532	23 522	19 207	34 012	30 575	34 919	41 128
	**Class II (*n* = 39)**	18 846	37 458	31 321	38 666	38 994	46 153	42 215	30 762
	**Class III (*n* = 33)**	26 746	21 885	33 898	43 587	43 378	50 763	42 750	35 187
**OA of the knee**	**Class I (*n* = 412)**	15 822	13 045	18 087	14 107	15 840	14 677	17 137	16 405
	**Class II (*n* = 259)**	17 345	15 692	13 351	12 497	15 510	12 197	17 836	18 134
	**Class III (*n* = 202)**	18 750	15 045	16 895	17 159	19 374	17 425	15 447	21 511
**High CV risk[a]**	**Class I (*n* = 2487)**	13 239	10 649	11 455	12 173	13 728	14 277	15 445	16 549
	**Class II (*n* = 1287)**	13 305	11 689	13 022	14 228	14 670	16 788	17 005	18 397
	**Class III (*n* = 917)**	13 661	11 988	13 600	15 113	15 541	15 654	18 155	19 899

**≥2 ORCs[b]**	**Class I (*n* = 4818)**	12 405	10 527	11 490	11 880	12 833	13 112	14 322	15 139
	**Class II (*n* = 2248)**	12 850	10 989	12 527	12 946	13 745	14 855	15 435	17 362
	**Class III (*n* = 1589)**	12 939	10 991	12 508	14 033	14 364	13 810	16 030	16 957
**≥3 ORCs[b]**	**Class I (*n* = 2387)**	15 692	12 810	14 426	14 188	16 353	16 408	18 550	19 220
	**Class II (*n* = 1179)**	15 640	13 261	14 320	15 272	16 389	18 366	19 214	20 916
	**Class III (*n* = 909)**	15 166	13 375	15 143	16 529	17 401	17 177	19 485	21 245

ASCVD atherosclerotic cardiovascular disease, *CKD* chronic kidney disease, *CVD* cardiovascular disease, *HF* heart failure, *OA* osteoarthritis, *ORC* obesity-related complication, *T2D* type 2 diabetes.

[a]High CV risk: ≥2 risk factors out of hypertension, dyslipidaemia and T2D/prediabetes.

[b]ORCs were obstructive sleep apnoea, HF, urinary incontinence, OA of the knee, T2D, prediabetes, asthma, psoriasis, gastro-oesophageal reflux disease, hypertension, dyslipidaemia, musculoskeletal pain, ASCVD and CKD.

Table S8 Observed mean inpatient, outpatient, ED and drug costs per person per year of follow-up for individuals with obesity.

Cost per person per year, 2019 US$		Baseline year	Year 1	Year 2	Year 3	Year 4	Year 5	Year 6	Year 7	Year 8
**T2D (*n* = 2611)**	**Inpatient costs**	2188	3497	2325	3114	3321	3952	3996	4058	4517
	**Outpatient costs**	4909	6909	6056	6556	6927	7417	7737	8784	8682
	**ED costs**	294	316	282	320	352	353	351	435	417
	**Drug costs**	3832	4351	4599	5012	5441	6112	6727	7222	7906
**Established CVD (*n* = 2091)**	**Inpatient costs**	7178	5013	3444	3163	3675	4770	4205	4341	5476
	**Outpatient costs**	8041	8380	7089	7456	7687	7697	8325	9571	9318
	**ED costs**	604	422	438	423	394	437	400	543	531
	**Drug costs**	3434	3810	3875	3985	4145	4291	4462	4806	5326
**HF (*n* = 197)**	**Inpatient costs**	19 420	11 053	7386	7758	6703	11 543	5562	6010	7590
	**Outpatient costs**	10 852	11 428	9286	10 418	11 472	9306	13 107	16 907	13 478
	**ED costs**	709	417	390	403	573	603	594	681	486
	**Drug costs**	3855	4226	4466	4586	4606	4838	4953	5727	5541
**CKD (*n* = 216)**	**Inpatient costs**	13 274	6873	6756	7752	6720	13 057	8474	7217	8205
	**Outpatient costs**	10 268	12 790	10 542	13 215	14 036	17 167	21 589	22 891	22 620
	**ED costs**	469	355	447	419	456	653	526	464	394
	**Drug costs**	4870	5031	5163	5549	5689	6119	6409	7325	7524
**OA of the knee (*n* = 873)**	**Inpatient costs**	4284	5371	4152	5297	3440	4674	3393	5129	4689
	**Outpatient costs**	7029	8281	6592	7262	6958	7717	6999	7107	8228

	**ED costs**	282	282	396	383	280	319	368	457	545
	**Drug costs**	2848	3299	3550	3847	3938	4169	4185	4716	5182
**High CV risk[a] (*n* = 4691)**	**Inpatient costs**	2745	3249	2109	2463	2719	3295	3298	3279	3958
	**Outpatient costs**	4937	6560	5458	5844	6321	6384	6932	7823	7934
	**ED costs**	333	283	271	282	325	314	321	402	370
	**Drug costs**	3148	3531	3629	3996	4272	4661	5005	5300	5819
**≥2 ORCs[b] (*n* = 8655)**	**Inpatient costs**	2606	2905	2044	2552	2753	3089	2894	3081	3487
	**Outpatient costs**	5442	6601	5476	5921	6192	6359	6671	7436	7755
	**ED costs**	361	318	321	324	329	332	335	418	404
	**Drug costs**	2765	3112	3212	3473	3606	3904	4128	4407	4808
**≥3 ORCs[b] (*n* = 4475)**	**Inpatient costs**	4037	3862	2667	3152	3147	3971	3939	4009	4618
	**Outpatient costs**	6739	7850	6378	7062	7273	7742	7985	9266	9336
	**ED costs**	446	358	352	368	372	375	391	505	479
	**Drug costs**	3514	3859	3998	4330	4529	4863	5156	5640	6124

ASCVD atherosclerotic cardiovascular disease, *CKD* chronic kidney disease, *CVD* cardiovascular disease, *ED* emergency department, *HF* heart failure, *OA* osteoarthritis, *ORC* obesity-related complication, *T2D* type 2 diabetes.

[a]High CV risk: ≥2 risk factors out of hypertension, dyslipidaemia and T2D/prediabetes.

[b]ORCs were obstructive sleep apnoea, HF, urinary incontinence, OA of the knee, T2D, prediabetes, asthma, psoriasis, gastro-oesophageal reflux disease, hypertension, dyslipidaemia, musculoskeletal pain, ASCVD and CKD.

Fig. S1 Observed mean annual total all-cause healthcare costs for individuals with obesity by number of ORCs, (a) averaged over 8 years follow-up, and (b) per year of follow-up.

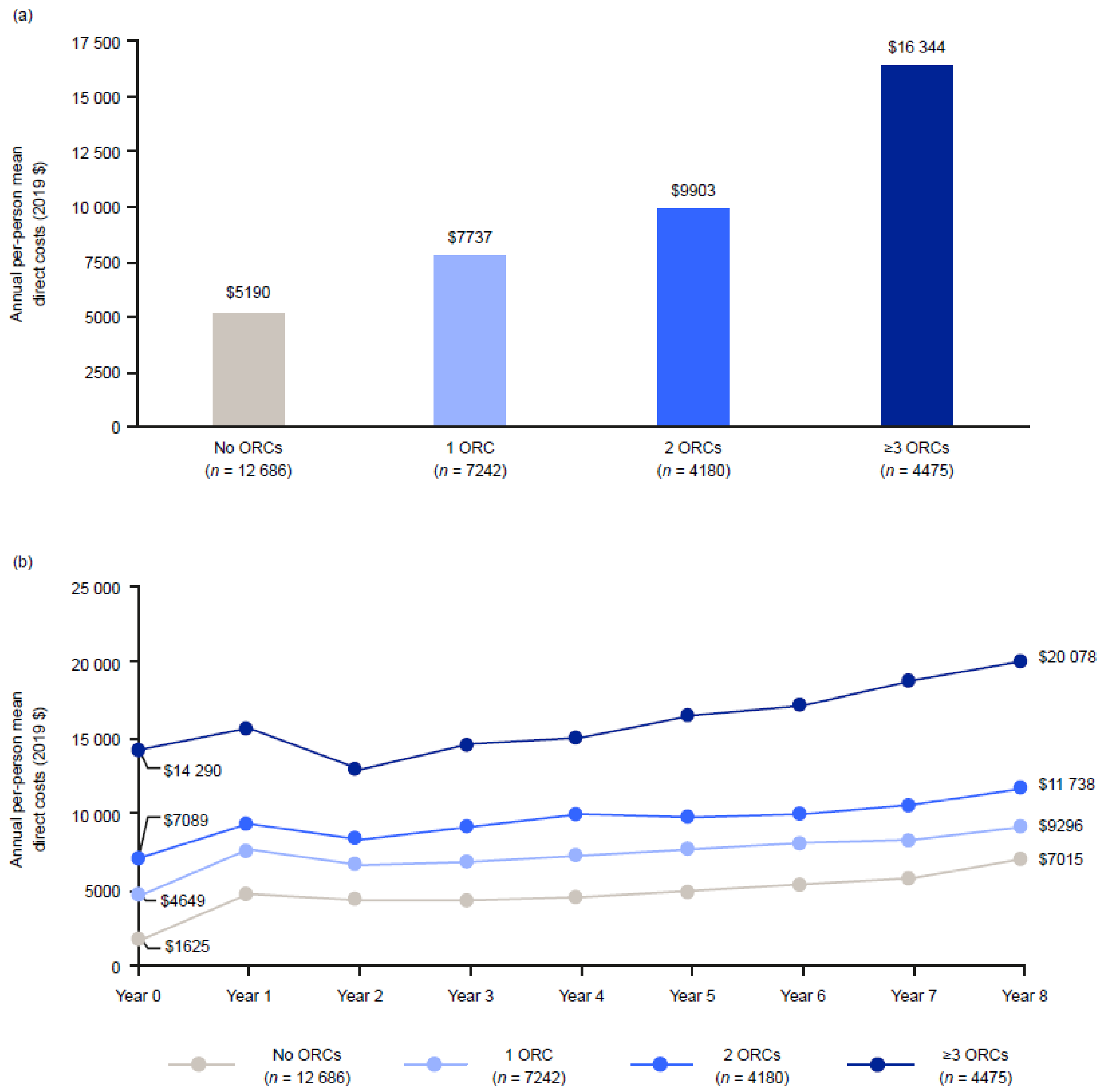

ORC obesity-related complication.

Fig. S2 Observed mean annual total all-cause healthcare costs for individuals with obesity, averaged over 8 years follow-up and stratified by the presence ('with' group) or absence ('without' group) of specific ORCs.

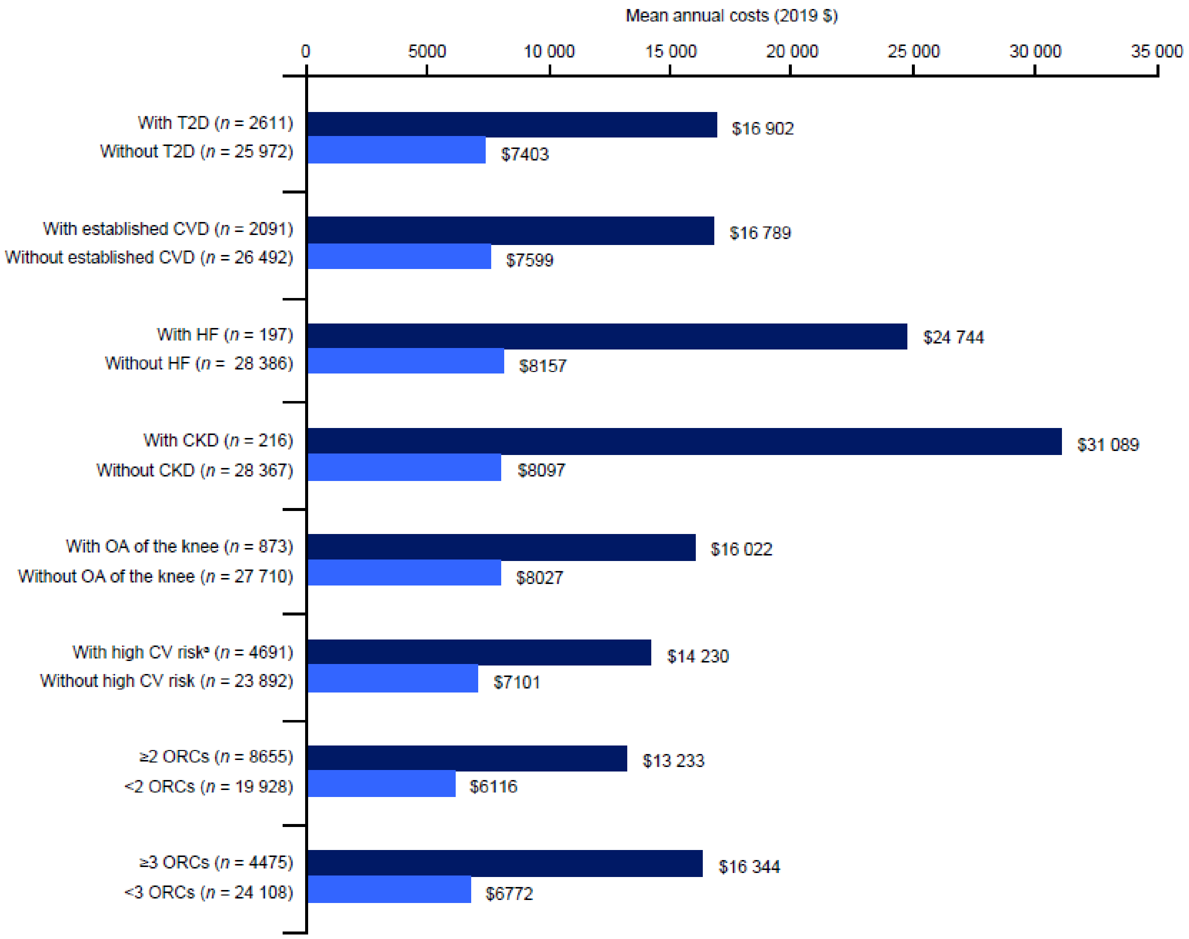

ASCVD atherosclerotic cardiovascular disease, *CKD* chronic kidney disease, *CV* cardiovascular, *CVD* cardiovascular disease, *HF* heart failure, *OA* osteoarthritis, *ORC* obesity-related complication, *T2D* type 2 diabetes.

[a]High CV risk: ≥2 risk factors out of hypertension, dyslipidaemia and T2D/prediabetes. The 'without' group has <2 CV risk factors.

Fig. S3 Observed mean total all-cause per-person healthcare costs at baseline, year 1 and year 8 among individuals with obesity, stratified by specific ORC.

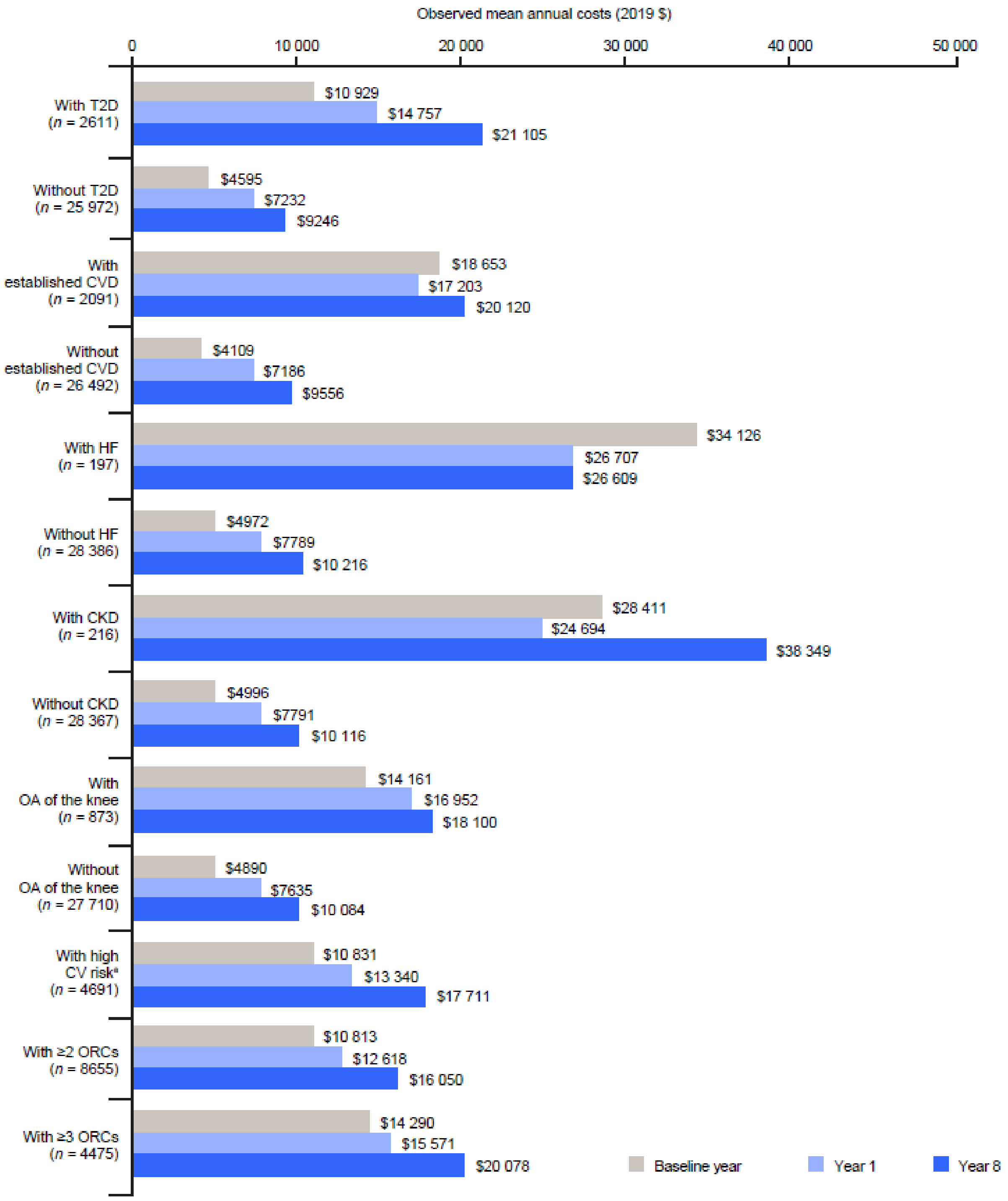

ASCVD atherosclerotic cardiovascular disease, *CKD* chronic kidney disease, *CV* cardiovascular, *CVD* cardiovascular disease, *HF* heart failure, *OA* osteoarthritis, *ORC* obesity-related complication, *T2D* type 2 diabetes.

[a]High CV risk: ≥2 risk factors out of hypertension, dyslipidaemia and T2D/prediabetes.
